# Parallels between retinal and brain pathology and response to immunotherapy in old, late‐stage Alzheimer's disease mouse models

**DOI:** 10.1111/acel.13246

**Published:** 2020-10-14

**Authors:** Jonah Doustar, Altan Rentsendorj, Tania Torbati, Giovanna C. Regis, Dieu‐Trang Fuchs, Julia Sheyn, Nazanin Mirzaei, Stuart L. Graham, Prediman K. Shah, Mitra Mastali, Jennifer E. Van Eyk, Keith L. Black, Vivek K. Gupta, Mehdi Mirzaei, Yosef Koronyo, Maya Koronyo‐Hamaoui

**Affiliations:** ^1^ Department of Neurosurgery Cedars‐Sinai Medical Center Maxine Dunitz Neurosurgical Research Institute Los Angeles CA USA; ^2^ College of Osteopathic Medicine of the Pacific Western University of Health Sciences Pomona CA USA; ^3^ Department of Clinical Medicine Macquarie University Sydney NSW Australia; ^4^ Save Sight Institute Sydney University Sydney NSW Australia; ^5^ Oppenheimer Atherosclerosis Research Center Cedars‐Sinai Heart Institute Los Angeles CA USA; ^6^ Department of Biomedical Sciences Cedars‐Sinai Medical Center Los Angeles CA USA; ^7^ Cedars‐Sinai Medical Center Smidt Heart Institute Los Angeles CA USA; ^8^ Barbara Streisand Women’s Heart Center Cedars‐Sinai Medical Center Los Angeles CA USA; ^9^ Department of Medicine Cedars‐Sinai Medical Center Los Angeles CA USA; ^10^ Department of Molecular Sciences Macquarie University Sydney NSW Australia; ^11^ Australian Proteome Analysis Facility Macquarie University Sydney NSW Australia

**Keywords:** astrocytes reactivation, glutamine synthetase, myeloid cells, neurodegenerative disease, ocular proteins, retina, synaptic preservation, vascular amyloidosis

## Abstract

Despite growing evidence for the characteristic signs of Alzheimer's disease (AD) in the neurosensory retina, our understanding of retina–brain relationships, especially at advanced disease stages and in response to therapy, is lacking. In transgenic models of AD (APP_SWE_/PS1_∆E9_; ADtg mice), glatiramer acetate (GA) immunomodulation alleviates disease progression in pre‐ and early‐symptomatic disease stages. Here, we explored the link between retinal and cerebral AD‐related biomarkers, including response to GA immunization, in cohorts of old, late‐stage ADtg mice. This aged model is considered more clinically relevant to the age‐dependent disease. Levels of synaptotoxic amyloid β‐protein (Aβ)_1–42_, angiopathic Aβ_1–40_, non‐amyloidogenic Aβ_1–38_, and Aβ_42_/Aβ_40_ ratios tightly correlated between paired retinas derived from oculus sinister (OS) and oculus dexter (OD) eyes, and between left and right posterior brain hemispheres. We identified lateralization of Aβ burden, with one‐side dominance within paired retinal and brain tissues. Importantly, OS and OD retinal Aβ levels correlated with their cerebral counterparts, with stronger contralateral correlations and following GA immunization. Moreover, immunomodulation in old ADtg mice brought about reductions in cerebral vascular and parenchymal Aβ deposits, especially of large, dense‐core plaques, and alleviation of microgliosis and astrocytosis. Immunization further enhanced cerebral recruitment of peripheral myeloid cells and synaptic preservation. Mass spectrometry analysis identified new parallels in retino‐cerebral AD‐related pathology and response to GA immunization, including restoration of homeostatic glutamine synthetase expression. Overall, our results illustrate the viability of immunomodulation‐guided CNS repair in old AD model mice, while shedding light onto similar retino‐cerebral responses to intervention, providing incentives to explore retinal AD biomarkers.

## Graphical abstract

In this study, Doustar et al. revealed that retinal Abeta burden predicts its brain levels in old, late‐stage murine models of Alzheimer's disease and further in response to immunotherapy. Substantial therapeutic effects are detected even at such advanced disease stage; immunomodulation effectively mitigates vascular and parenchymal amyloid‐beta deposition, diminishes neuroinflammation, as well as restores synaptic density and retino‐cerebral glutamine synthetase levels.

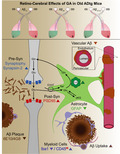

## 1 INTRODUCTION

In recent years, a surge of studies have indicated that the pathological hallmarks of Alzheimer's disease (AD) extend beyond the brain and manifest in the retina of patients (Alexandrov et al., 2011; den Haan et al., 2018; Hadoux et al., 2019; Koronyo et al., 2017; Koronyo‐Hamaoui et al., 2011; La Morgia et al., 2016; Schon et al., 2012; Schultz et al., 2020; Shi et al., 2020; Tsai et al., 2014). AD is a fatal neurodegenerative disorder and the most common form of dementia clinically diagnosed by the progressive loss of memory and cognitive function (Alzheimer's Association, 2018). Neuropathologically, AD is typified by progressive, age‐dependent accumulation of amyloid β‐protein (Aβ; Sperling et al., 2020) in both soluble and insoluble plaque conformations, as well as neurofibrillary tangles mostly comprised of hyperphosphorylated tau, leading to vast synaptic and neuronal degeneration (De Strooper & Karran, 2016; Hardy et al., 1998; Li et al., 2020). In particular, Aβ_42_ and Aβ_40_ alloforms have been strongly implicated in AD pathogenesis and cognitive decline (Benilova et al., 2012; Chiti & Dobson, 2017; Kayed et al., 2003; Lambert et al., 1998; McLean et al., 1999; Shankar et al., 2008). Neuroinflammation is another key component of the AD pathological continuum characterized by a marked presence of chronically activated microglia (microgliosis) and reactive astrocytes (astrocytosis) in the vicinity of Aβ deposition. These are commonly viewed as detrimental immune responses in the AD brain and have been linked to synaptic loss and cognitive decline (Heneka et al., 2015; Wyss‐Coray & Mucke, 2002).

To reduce amyloidosis and regulate neuroinflammation, various immunomodulatory strategies were investigated for their potential therapeutic impact in preclinical rodent models of AD (Bakalash et al., 2011; Bernstein, et al., 2014; Butovsky et al., 2006, 2007; Frenkel et al., 2005; Koronyo et al., 2015; Koronyo‐Hamaoui et al., 2009, 2011, 2014, 2020; Li et al., 2020; Rentsendorj et al., 2018; Rosenzweig et al., 2019). Many recent studies have demonstrated beneficial effects of these immunomodulatory approaches in preventing, halting, or reversing AD pathologies and preserving memory and learning functions. Glatiramer acetate (GA; also termed Copaxone^®^), an FDA‐approved drug used for the treatment of remitting‐relapsing multiple sclerosis, has displayed promising results in young and adult double‐transgenic APP_SWE_/PS1_∆E9_ (ADtg) mice. Aβ burden, astrocytosis, and microgliosis were diminished, brain milieu shifted from a pro‐ to anti‐inflammatory profile, synapses were rescued, and hippocampal neurogenesis was induced, ultimately preventing cognitive decline (Bakalash et al., 2011; Baruch et al., 2015; Butovsky et al., 2006, 2007; Frenkel et al., 2005; Koronyo et al., 2012, 2015; Rentsendorj et al., 2018). The therapeutic mechanisms of GA immunization, mainly explored in these transgenic AD model mice (Butovsky et al., 2006, 2007; Koronyo et al., 2012, 2015; Rentsendorj et al., 2018), were attributed to a shift in microglial phenotype and enhanced recruitment of neuroprotective, peripherally derived monocytes and macrophages directly involved in Aβ clearance, immunoregulation, and neuroregeneration (Koronyo et al., 2015; Rentsendorj et al., 2018). While these promising effects were reported in pre‐ and early‐symptomatic (5‐ to 12‐month‐old) ADtg mice, they have never been investigated in old, late‐stage mouse models of AD. Given that aging is a fundamental factor in AD development (Sperling et al., 2020), mice allowed to progress into old age may better represent the clinical manifestations of human AD. Such old mice could offer greater insights into the potential translation of effective therapies.

Beyond the brain, a growing number of studies have provided evidence for AD‐specific protein aggregation, vascular pathology, and markers of neuroinflammation in the neurosensory retina of various transgenic, induced, and spontaneous animal models of AD (Chang et al., 2020; Chiasseu et al., 2017; Do et al., 2019; Doustar et al., 2017; Grimaldi et al., 2018; Hampel et al., 2018; Hart et al., 2016; Koronyo et al., 2012; Lei et al., 2017), as well as in human AD patients (Alexandrov et al., 2011; den Haan et al., 2018; Hadoux et al., 2019; Koronyo et al., 2017; Koronyo‐Hamaoui et al., 2011; La Morgia et al., 2016; Schon et al., 2012; Schultz et al., 2020; Shi et al., 2020; Tsai et al., 2014). Biochemical analyses of Aβ_40_ and Aβ_42_ peptide levels in retinal and brain tissues from several transgenic murine models and AD patients revealed increases in both peptides in the AD retina as compared to controls, with higher levels in the brain and correlations with brain levels (La Morgia et al., 2016; Schultz et al., 2020; Shi et al., 2020). However, assessment of such relationships between paired retinas derived from oculus sinister (OS) versus oculus dexter (OD) eyes and left versus right cerebral hemispheres for Aβ load was not previously undertaken. This is especially true for response to therapy and in old ADtg mice. A recent study showed the accumulation of Aβ_40_ and Aβ_42_ peptides in whole ocular tissues of 5× FAD transgenic mice with a reduction in ocular Aβ following neprilysin treatment (Parthasarathy et al., 2015). Although these findings are significant, the inclusion of non‐neuronal tissue and the lack of analyzed brain tissues hinder the ability to evaluate connections between the neuro‐retina and brain. In this context, we previously demonstrated a comparable Aβ‐deposit reduction in retinal and cerebral tissues of 12‐month‐old ADtg mice subjected to immunomodulation with MOG‐45D (altered myelin‐derived peptide) loaded on dendritic cells (Koronyo‐Hamaoui et al., 2009, 2011) and subsequently showed the feasibility to noninvasively detect progressive appearance and clearance of individual retinal Aβ plaques following GA immunization (Koronyo et al., 2012).

Collectively, these early studies provide the rationale to explore the relationship between retinal and cerebral pathology, including accumulation of both non‐amyloidogenic and disease‐associated amyloidogenic Aβ alloforms, in old age and later stages of disease. There is also a need to study the responses to immunomodulation intervention in more clinically relevant, aged murine models and to quantitatively determine parallels between the brain and retina. Addressing the above unknowns, this study provides evidence for the predictability of cerebral Aβ_42_ and Aβ_40_ burden via quantitative measurements of OS and OD retinal counterparts in old ADtg mice. Moreover, in addition to identifying similar responses in the neuro‐retina and the brain this study demonstrates the efficacy of GA immunomodulation in restricting vascular pathology and neuroinflammation while improving synaptic density at such late‐stage disease.

## 2 RESULTS

### 2.1 Lateralization of Aβ levels in retinae and brain hemispheres from old AD model mice

**Figure 1:**
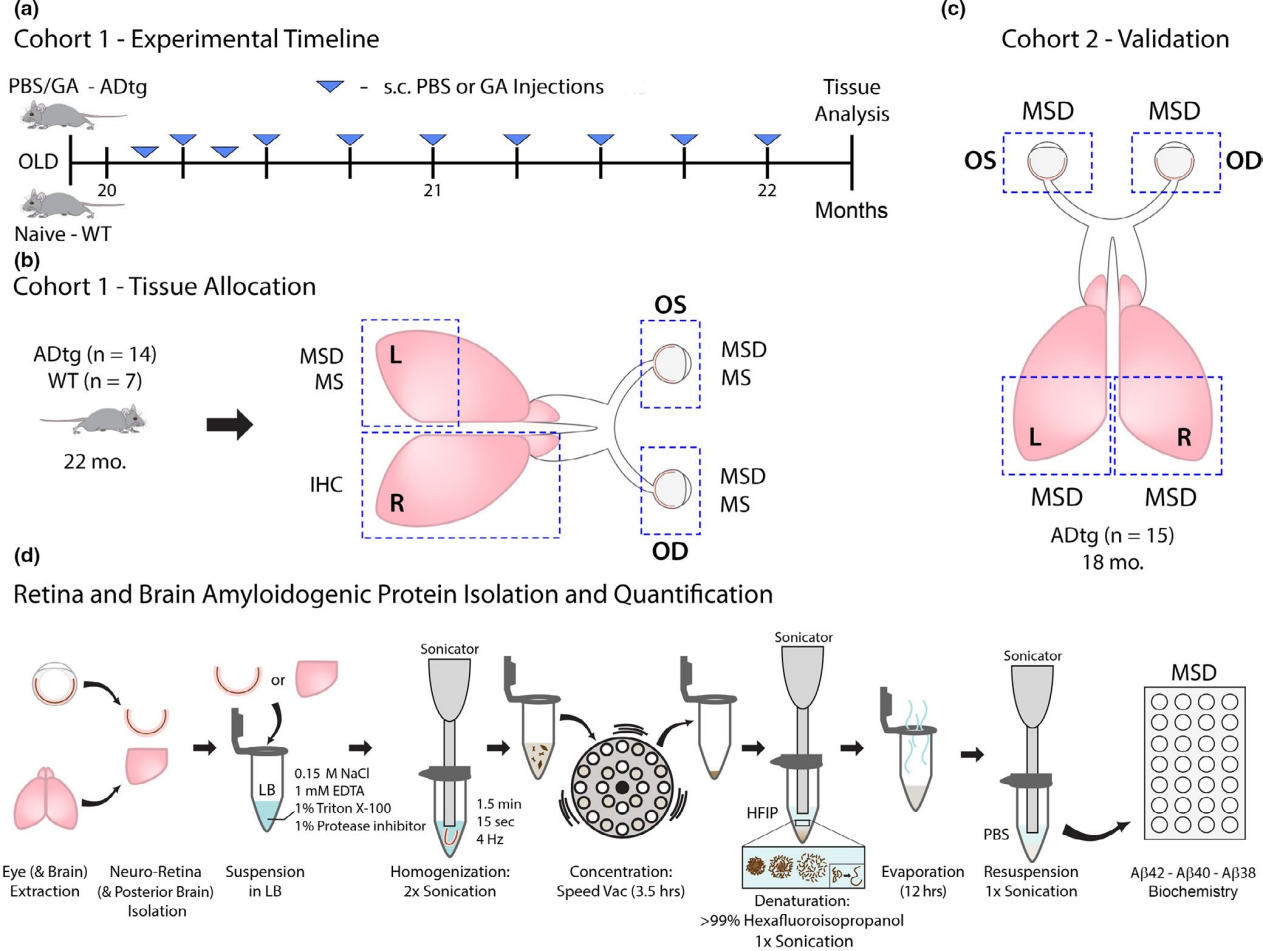
Experimental design and intervention timeline assessing cerebral and retinal tissues in old, late‐stage ADtg mice. (a) Experimental timeline for mouse Cohort 1: 20‐month‐old APP_SWE_/PS1_ΔE9_ (ADtg) mice underwent weekly, subcutaneous injections of glatiramer acetate (GA, also known as Copaxone^®^; 100 µg) for a 2‐month duration (*n* = 7 mice). Age‐ and sex‐matched ADtg mice were subcutaneously injected with PBS in the same regimen and naïve non‐transgenic (WT) littermates were used as controls (*n* = 7 mice per group). (b) One week following the last injection, mice were sacrificed, and tissues were collected as described. Paired brains and eyeballs from Cohort 1 were allocated for further analyses: the right (R) cerebral hemisphere was used for immunohistochemistry (IHC), the left (L) posterior brain was used for quantitative biochemical Meso Scale Discovery (MSD) and Mass Spectrometry (MS) assays, and both oculus sinister (OS, left) and oculus dexter (OD, right) eyeballs were collected, the neurosensory retinae isolated, and proteins assessed by MSD and MS analyses. OS and OD retinae were separately analyzed by MSD and pooled together for MS analysis. (c) Cohort 2 was comprised of old ADtg mice (*n* = 15 mice; average age of 18 months). L and R posterior brains as well as OS and OD eyeballs were collected and analyzed separately for Aβ proteins by MSD. (d) Preparation of mouse neuro‐retina and posterior brain Aβ proteins for quantification by MSD. Each tissue was prepared separately for analysis (OS retina, OD retina, and left and right posterior brains). The protocol involves suspension in lysis buffer (LB), homogenization via sonication, concentration with speed vac, and protein denaturation with hexafluoroisopropanol (HFIP) followed by evaporation and resuspension in PBS prior to protein concentration analysis

To investigate levels of disease‐associated amyloidogenic (Aβ_1–42_ and Aβ_1–40_) and non‐amyloidogenic (Aβ_1–38_) alloforms in neurosensory retina and brain tissues, as well as evaluate the relationship between retinal and brain Aβ burden, we analyzed two cohorts of 18‐ and 22‐month‐old, late‐stage transgenic APP_SWE_/PS1_∆E9_ (ADtg) mice (Figure 1a–c). Further, to assess retinal and brain Aβ pathology in response to immunotherapy in old mice, a cohort of 20‐month‐old ADtg mice underwent GA immunization and were compared against age‐ and sex‐matched PBS‐control ADtg and naive WT mice (Cohort 1, experimental timeline and GA immunization regimen are shown in Figure 1a; *n* = 7 mice per group). Cohort 2 composed of old, untreated 18‐month‐old ADtg mice (*n* = 15) were allotted to validate concentrations of Aβ alloforms in retinal and brain tissues. Retinae derived from OS and OD eyes, as well as left and right posterior brains, were allocated for histological (immunohistochemistry—IHC) or biochemical (Meso Scale Discovery—MSD, mass spectrometry—MS) analyses as outlined in Figure 1b–c. To this end, we established a new protocol encompassing extraction, enrichment, and hexafluoroisopropanol (HFIP)‐mediated unfolding of Aβ proteins (Figure 1d).

**Figure 2:**
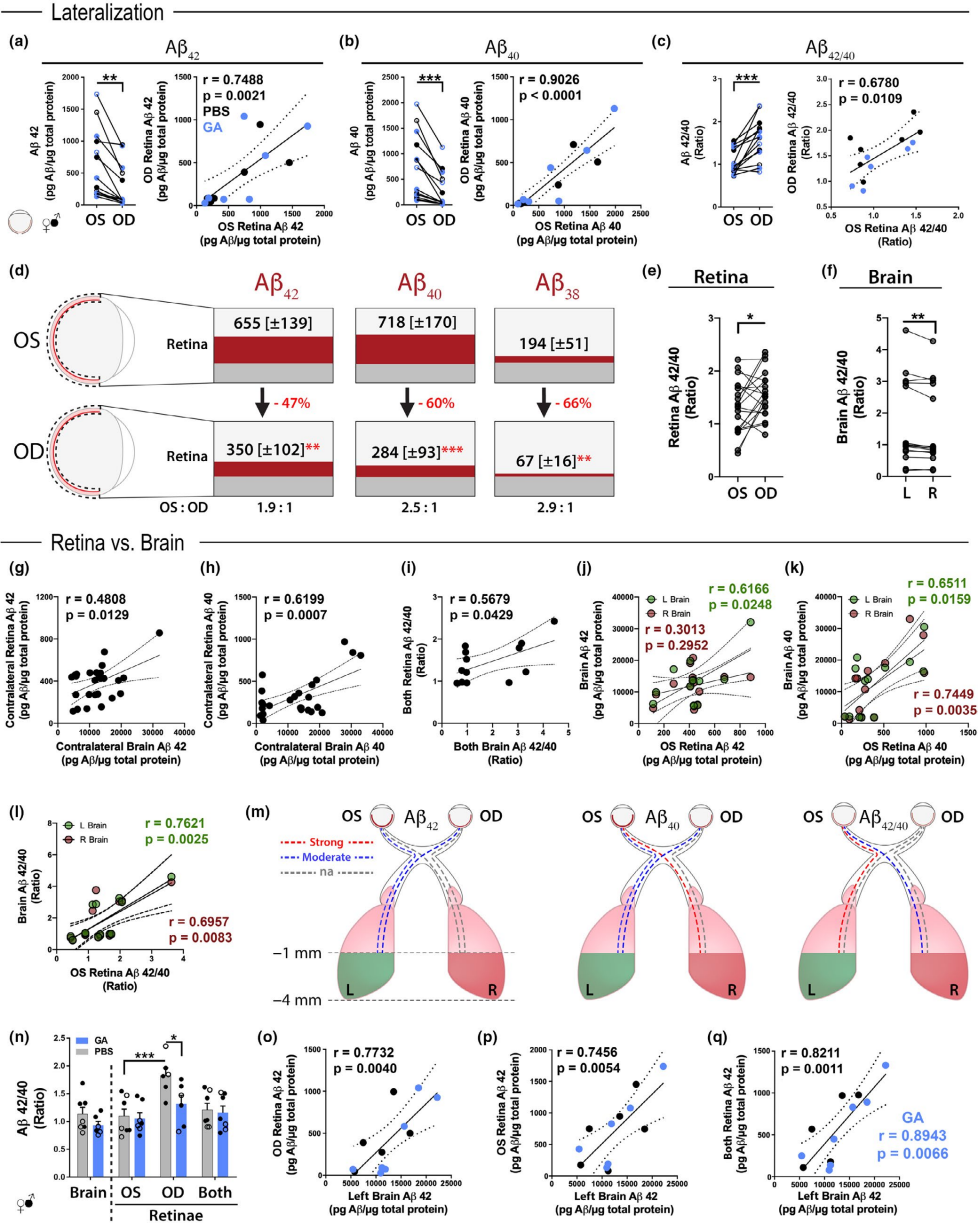
Retinal and cerebral Aβ alloforms in old ADtg mice and following immunotherapy. (a–c) Analysis of Aβ_1–42_, Aβ_1–40,_ and Aβ_42/40_ ratio levels (*n* = 7 mice per group) in OS versus OD retinae from Cohort 1 of GA‐immunized (blue) and PBS‐control (black) old ADtg mice. Data indicate Aβ concentrations for individual mouse in OS versus OD retina analyzed by paired Student's *t test*. Pearson's *r* correlations between levels of each Aβ_1–42_, Aβ_1–40_, and Aβ_42/40_ ratio in OS and OD retinae are also shown (*n* = 13–14 mice; right graphs). (d) Schematic display of Aβ_1–42_, Aβ_1–40_, and Aβ_1–38_ alloform concentrations (Average; ± SEM in brackets) in each retina from Cohort 1. Data presented in pg Aβ per µg total protein. Lower percentages of amyloid levels in OD versus OS retinae are shown in red, and OS to OD ratios of Aβ concentrations are indicated below. (e–f) Aβ_42/40_ ratios as assessed by MSD analysis in paired OS versus OD retinae from ADtg mice (e; Cohorts 1 and 2 without GA group; *n* = 20 mice) and in paired L versus R brains from Cohort 2 ADtg mice (f; *n* = 15 mice). Lateralization was determined by paired Student's *t test*. (g–m) Pearson's *r* correlations between retinal and cerebral Aβ burden in ADtg mice from Cohort 2. (g–h) Contralateral correlations between OS retina versus R brain and OD retina versus L brain for (g) Aβ_42_ and (h) Aβ_40_ levels (*n* = 12–14). (i) Correlation between average retinal and average cerebral Aβ_42/40_ ratios. (j–l) Unilateral correlations of OS retina versus L brain (green) or versus R brain (red) for (j) Aβ_42_, (k) Aβ_40_, and (l) Aβ_42/40_ ratio (*n* = 13). (m) Schematic illustration portraying strength of associations between each retina and each posterior brain for Aβ_42_, Aβ_40_, and Aβ_42/40_ ratio in old ADtg mice. The analyzed posterior brain includes tissue between −1 and −4 mm bregma. Strong associations in red (*r* > 0.7), moderate associations in blue (*r* = 0.5–0.7), and weak or no associations (na) in gray (*r* = 0.0–0.5). (n–q) Analysis of retinal versus cerebral Aβ levels in Cohort 1 old ADtg mice following GA immunization. (n) Brain and retinal Aβ_42/40_ ratios in PBS‐control versus GA‐immunized ADtg mice (*n* = 7 mice per group). (o–q) Pearson's *r* correlations between levels of Aβ_42_ in L brain and (o) OD retina, (p) OS retina, and (q) an average of both retinae (*n* = 14 mice). Strong correlations in Aβ_1–42_ burden between brain and retinal tissues are especially apparent following GA immunization (q). Graphs display individual data point for each mouse, with bar graphs also indicating group mean and standard error of mean (SEM) values. Mouse sex is designated as filled circles for males and open circles for females (gender not shown in correlation graphs). *p < 0.05, **p < 0.01, ***p < 0.001 assessed by paired Student's *t* test for two‐group comparisons, and a two‐way ANOVA with Sidak's post‐test for group analysis for brain and retinal tissues

Levels of Aβ_42_, Aβ_40_, and Aβ_38_ alloforms measured by MSD in paired OS and OD retinae of PBS‐control and GA‐immunized ADtg mice (Cohort 1) are displayed in Figure 2a–b (extended data in Figure S1A–C). Another principle characteristic of AD neuropathology is the ratio between Aβ_42_ and Aβ_40_. We further calculated and compared Aβ_42/40_ ratios between OS and OD retinae in this cohort (Figure 2c). Consistent disparities in Aβ alloform accumulation between paired OS and OD retinae were found, with significantly higher levels in the OS versus the OD retina in old transgenic mice (Aβ_42_, *p* = 0.0015; Aβ_40_, *p* = 0.0005; and Aβ_38_, *p* = 0.0063, by paired two‐tailed Student's *t* test; Figures 2a–b and S1A). Lateralization of Aβ alloforms was independent of treatment group and gender (females: Aβ_42_, *p* = 0.0346; Aβ_40_, *p* = 0.0209; Aβ_38_, *p* = 0.0280; males: Aβ_42_, *p* = 0.0056; Aβ_40_, *p* = 0.0122; Aβ_38_, *p* = 0.2036; by paired two‐tailed Student's *t* test). Interestingly, paired analysis of Aβ_42/40_ ratio similarly showed lateralization, albeit higher in OD versus OS retina (*p* = 0.0004, paired two‐tailed Student's *t* test; Figure 1c). Despite this asymmetry, tight correlations in levels of Aβ alloforms and Aβ_42/40_ ratios were revealed between paired OS and OD retinae (Pearson's correlation coefficient *r* = 0.75 and *p* = 0.0021 for Aβ_42_, *r* = 0.90 and *p* < 0.0001 for Aβ_40_, *r* = 0.94 and *p* < 0.0001 for Aβ_38_, *r* = 0.68 and *p* = 0.0109 for Aβ_42/40_; Figures 2a–c and S1A). Figure 2d illustrates the average (±SEM) Aβ burden in OS versus OD retinae (655 ± 139 vs. 350 ± 102 for Aβ_42_; 718 ± 170 vs. 284 ± 93 for Aβ_40_; 194 ± 51 vs. 67 ± 16 for Aβ_38_, respectively; pg Aβ per µg total protein). Aβ levels are 47%–66% lower in the OD retina as compared to their corresponding levels in the OS retina in this old mouse model (Figure 2d). Additional data on retinal Aβ pathology from Cohort 1 are included in Figure S1B–F.

In light of the significant disparities in Aβ levels between OS versus OD retina, we aged an additional mouse Cohort 2 (18‐month‐old ADtg mice) and assessed lateralization of Aβ burden in paired retinae and brains at advanced disease stage without intervention (Figure 2e–f; extended data in Figure S1G–L). MSD results from combined Cohorts 1 and 2 validated a significant OS retina dominant lateralization for Aβ_42_ and Aβ_40_ levels (*p* = 0.0057 and *p* = 0.0082, *r*espectively, by two‐tailed paired Student's *t* test; *n* = 21, Figure S1G–H). Retinal Aβ_38_ alloforms were below detectable levels for most retinae from this cohort. As it refers to disease‐relevant Aβ_42/40_ ratios, OD retina dominant lateralization was identified in these old ADtg mice (*p* = 0.0335, by two‐tailed paired Student's *t* test; Figure 2e). In agreement with the above results, positive correlations were found between levels of Aβ_42_, Aβ_40_, and Aβ_42/40_ in OS versus OD retinae (Figure S1G–I). Correlations between OS and OD retinae for Aβ_42_ levels were consistently strong (Figure S1G; Pearson's *r* = 0.7123 and *p* = 0.0003).

These findings in OS versus OD retinae led to the question of whether lateralization in Aβ levels also exists in the brains of old ADtg mice. Examination of bilateral brain tissues from Cohort 2 for Aβ alloforms by MSD analysis revealed left posterior brain‐dominant lateralization in Aβ_42/40_ ratios (*p* = 0.0061, paired two‐tailed Student's *t* test; *n* = 15; Figure 2f, extended data in Figure S1J–L). Although no one‐side (left or right) dominance of posterior brain lateralization was found for individual Aβ_42_, Aβ_40_, and Aβ_38_ alloforms, consistent disparities in their levels were obtained between the two hemispheres (Figure S1J–K; data not shown for Aβ_38_). Similar to the retina, all Aβ alloform levels significantly correlated between left and right posterior brains (Pearson's *r* = 0.59 and *p* = 0.0279 for Aβ_42_; *r* = 0.76 and *p* = 0.0009 for Aβ_40_; *r* = 0.68 and *p* < 0.0100 for Aβ_38_; Figure S1J–K). An especially strong correlation was found for levels of Aβ_42/40_ ratio (*r* = 0.98 and *p* < 0.0001; Figure S1L). Moreover, levels of each amyloidogenic Aβ_42_ and Aβ_40_ alloform strongly predicted co‐accumulation in the same retina or brain location (Pearson's *r* > 0.90 and *p* < 0.0001 across all retinal and brain tissues; Figure S2A–F).

### 2.2 Association between retinal and brain Aβ levels

Next, to determine the feasibility of predicting cerebral Aβ burden via its levels in the retina, brain levels were correlated against contralateral or ipsilateral, each OS and OD, and both retinae in old ADtg mice without intervention (Figures 2g–m and S2G–R and S3A‐L; Cohort 2). This assessment revealed that at advanced disease stage, significant correlations exist between levels in contralateral retina and brain tissues (OS retina to right brain or OD retina to left brain) for both amyloidogenic alloforms (Pearson's *r* = 0.48 and *p* = 0.0129 for Aβ_42_, *r* = 0.62 and *p* = 0.0007 for Aβ_40_; *n* = 26; Figure 2g–h; extended data on ipsilateral and contralateral correlations for each Aβ alloform and Aβ_42/40_ ratios in Figure S2G–J). Cumulative peptide values for both retina and both brains from each mouse were compared, indicating significant correlations of retinal to brain Aβ_42/40_ ratios and Aβ_40_ levels (Pearson's *r* = 0.57, *p* = 0.0429 and *r* = 0.59, *p* = 0.0328, respectively; *n* = 13; Figures 2i and S2L), and a significant correlation of OS retinal Aβ_42_ against both brain Aβ_42_ levels (*r* = 0.595 and *p* = 0.0321; Figure S2M). Individual correlations between OS or OD retina compared to paired left or right brain for each Aβ alloform demonstrated the greatest degree of associations between OS retina to left brain for Aβ_42_ (Pearson's *r* = 0.62 and *p* = 0.0248; Figure 2j) and OS retina to left and right brains for Aβ_40_ (*r* = 0.65 and *p* = 0.0159 for left and *r* = 0.74 and *p* = 0.0035 for right brain; Figure 2k). Similarly, most significant correlations for Aβ_42/40_ ratios were identified for OS retina to left and right brains (*r* = 0.76 and *p* = 0.0025 for left and *r* = 0.696 and *p* = 0.0083 for right brain; *n* = 13; Figure 2l). Figure 2m illustrates all individual Pearson's (*r*) correlations, with blue and red dashed lines designating moderate to strong significant associations (extended data in Figure S3A–L).

### 2.3 Retinal and cerebral Aβ levels strongly correlate in response to intervention

To evaluate effects of GA immunization on levels of retinal and brain Aβ alloforms in old ADtg mice (Cohort 1), we initially compared MSD data between GA‐immunized and PBS‐control groups (Figures 2n and S1D–F). Both the retinae and the posterior left brain did not show significant reductions in Aβ levels following GA immunization at this advanced, late‐stage disease (Figure S1D–F). However, Aβ_42/40_ ratio levels in the OD retina were significantly reduced in GA‐immunized as compared to PBS‐control mice (*p* = 0.0246, two‐way ANOVA with Sidak's post‐test; Figure 2n). Despite marked differences between brain and retina levels for each Aβ alloform (Figure S1D–F), very similar Aβ_42/40_ ratios were detected across the two CNS tissues, with the exception of higher ratio in the OD retina in PBS‐control ADtg mice (*p* = 0.0007; by two‐way ANOVA and Sidak's post‐test; Figure 2n).

To determine possible connections between retino‐cerebral Aβ levels in response to GA immunization, levels of Aβ_42_, Aβ_40,_ and Aβ_38_ alloforms in the left posterior brain were correlated against OD, OS, or both retinae in GA‐immunized and control mice (Figures 2o–q and S3M–R; Cohort 1). Strong linear correlations were identified between cerebral and OD, OS, and both retinal Aβ_42_ levels (*r* = 0.77 and *p* = 0.004, *r* = 0.746 and *p* = 0.0054, *r* = 0.82 and *p* = 0.0011, respectively; *n* = 13; Figure 2o–q). These results are in accordance with retinal Aβ_42_ levels' correlation with the left posterior brain, as described for untreated old ADtg mice above (Cohort 2; Figure 2j). Importantly, a separate Pearson's *r* analysis for GA‐immunized ADtg mice revealed an even stronger association between retinal and cerebral Aβ_42_ loads (*r* = 0.89 and *p* = 0.0066; Figure 2q, in blue). Retinal and brain correlations for Aβ_40_ or non‐amyloidogenic Aβ_38_ levels were weaker, reaching statistical significance between OD/OS retina to left posterior brain for Aβ_40_ and Aβ_38_ (*r* = 0.58 and *p* = 0.0391 and *r* = 0.58 and *p* = 0.0499, respectively; Figure S3M–R).

### 2.4 Immunomodulation ameliorates vascular and parenchymal Aβ deposits in old ADtg mice

**Figure 3:**
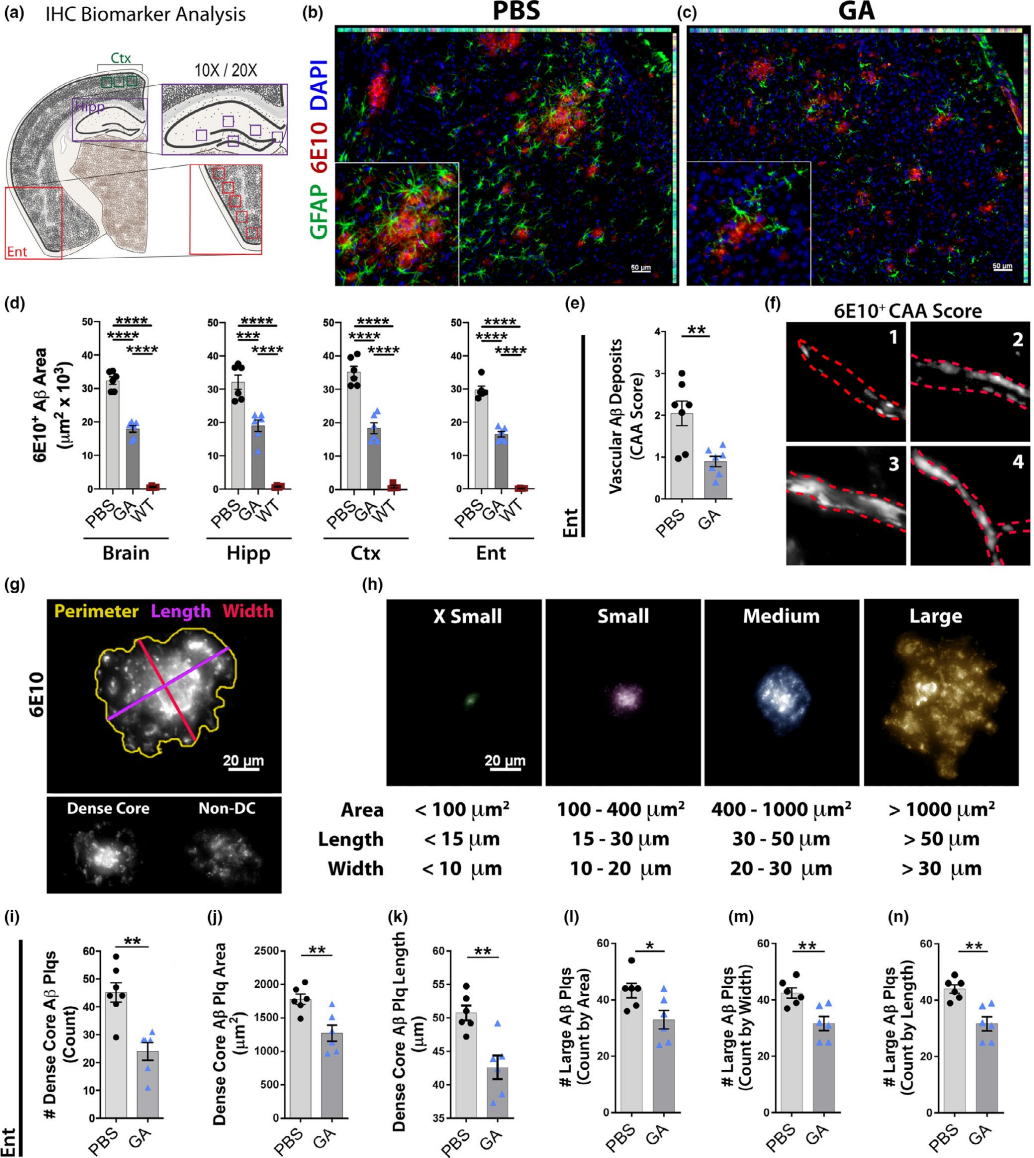
Decreased cerebral Aβ ‐plaque pathology and vascular amyloidosis in old ADtg mice following GA immunotherapy. (a) Cerebral map indicating specific brain regions analyzed by IHC; regions include the cingulate cortex/retrosplenial area (Ctx), hippocampus (Hipp), and entorhinal cortex/piriform area (Ent). (b–c) Representative coronal sections of a cortical region stained for astrocytes (GFAP, green), Aβ plaques (6E10, red), and cell nuclei (DAPI; blue) in (b) PBS‐control and (c) GA‐treated ADtg mice. (d) Quantitative IHC analysis of 6E10^+^ Aβ‐plaque area in total brain, Hipp, Ctx, and Ent of all experimental groups (*n* = 6 mice per group). (e) Analysis of cerebral amyloid angiopathy (CAA) scores in the Ent of GA‐immunized versus untreated (PBS) old ADtg mice (*n* = 7 mice per group). (f) Representative images illustrating the scoring method used to assess vascular 6E10^+^ Aβ deposits [termed as cerebral amyloid angiopathy (CAA) scores], with scale ranges from 0 to 4, with higher scores for greater vascular Aβ pathology. (g) Representative images and measurements of perimeter, length (largest diameter), and width (smallest diameter) acquired per Aβ plaque (top); Dense‐core and non‐dense‐core/diffuse plaque subtypes are demonstrated (bottom). (h) Microscopic images showing classification of plaque by size, as defined by area, length and width. Accordingly, plaques were categorized into four subgroups: x‐small, small, medium, and large. (i–k) Quantitative analysis of dense‐core plaque phenotype within the Ent of GA‐immunized versus PBS‐control ADtg mice, including (i) total count, (j) average area, and (k) length. (l–n) Quantitative analysis of large Aβ‐plaque count, as determined by (l) area, (m) width, and (n) length, within the Ent of GA‐immunized versus PBS‐control ADtg mice (*n* = 6–7 mice per group). Bar graphs indicate mean, standard error of mean (SEM), and individual data points. *p < 0.05, **p < 0.01, ***p < 0.001, ****p < 0.0001 assessed by unpaired Student's *t* test for two‐group comparisons, and a one‐way ANOVA with Tukey's post‐test for three or more groups

**Figure 4:**
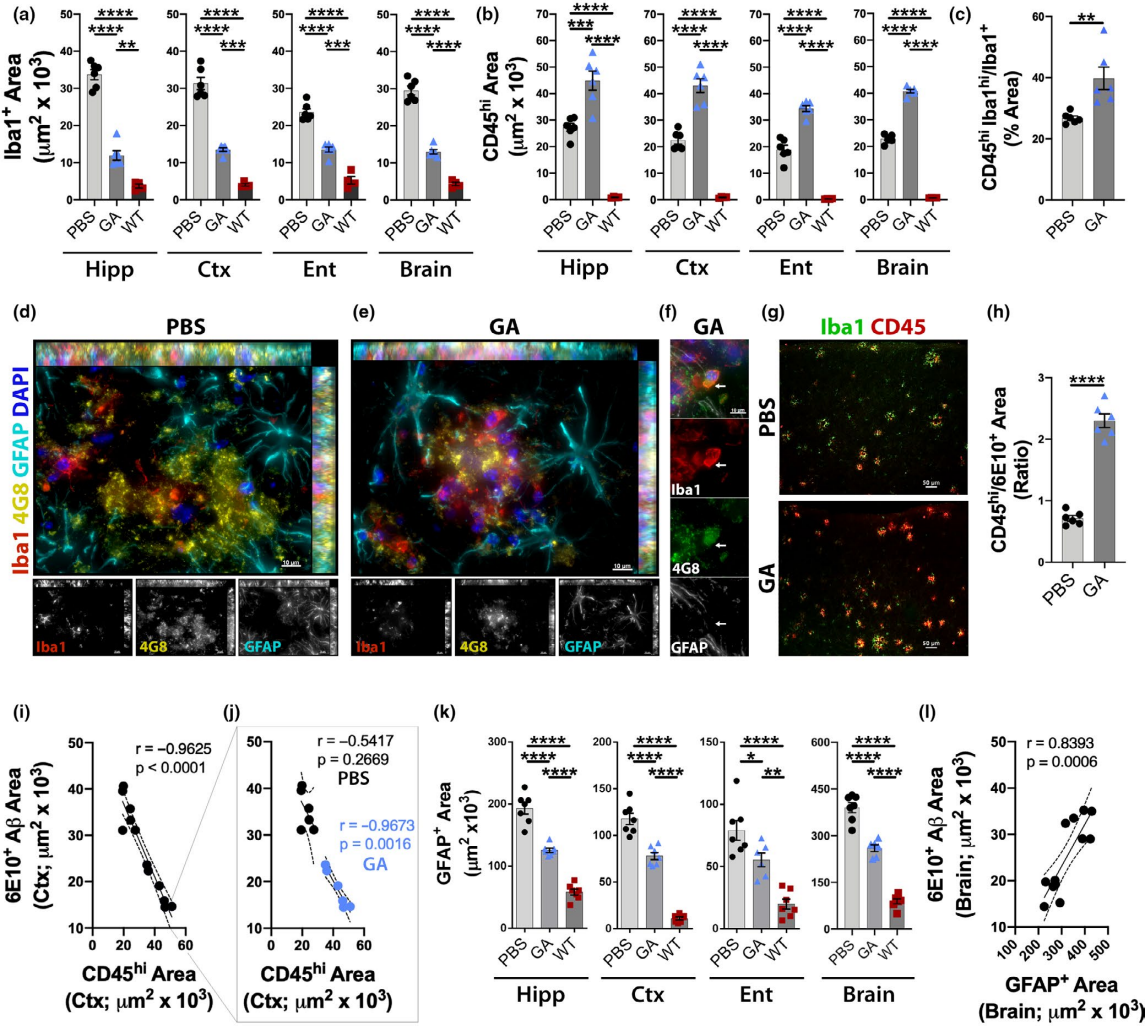
Reduced cerebral microgliosis and astrogliosis along with peripheral monocyte recruitment following immunomodulation. (a) Quantitative IHC analysis of Iba1^+^ microgliosis within predetermined brain regions: Hipp, Ctx, and Ent, as well as their average (Brain) in GA‐immunized versus PBS‐control ADtg mice, and in naïve WT mouse littermates. (b) Quantitative IHC analysis of Iba1^+^CD45^hi^ infiltrating peripheral immune cells in brain regions and their average (Brain) (*n* = 6 mice per group). (c) Analysis of percent area of Iba1^hi^/CD45^hi^ peripheral monocytes population of Iba1^+^ myeloid cell area within PBS‐control and GA‐immunized groups (*n* = 6 mice per group). (d–e) Representative micrographs of inflammatory cells, GFAP^+^ astrocytes (cyan) and Iba1^+^ myelomonocytes (red), surrounding 4G8^+^ Aβ plaques (yellow) in the Ent cortex of old, late‐stage (d) PBS‐control and (e) GA‐immunized ADtg mice. (f) A representative high‐magnification micrograph of Ent cortex of GA‐immunized ADtg mice with an Iba1^+^ myelomonocytic cell (red) seen engulfing 4G8^+^ Aβ; white arrow tags location between channels. (g) Representative micrographs of Iba1^+^ myelomonocytes and CD45^+^ hematopoietic immune cells within PBS‐control and GA‐immunized mice. (h) IHC analysis of CD45^hi^ area/6E10^+^ area ratio (*n* = 6 mice/group). (i) Pearson's *r* correlation analysis between CD45^hi^ hematopoietic cells and 6E10^+^ Aβ‐plaque deposits in Ctx sections including PBS‐control and GA‐immunized mice (*n* = 12 mice). (j) Separate Pearson's *r* correlations between CD45^hi^ hematopoietic cells and 6E10^+^ Aβ‐plaque deposits in old PBS‐control and GA‐immunized ADtg mice demonstrating retention of correlation with treatment. (k) Quantitative IHC analysis of GFAP^+^ astrogliosis in total brain regions for all experimental groups (*n* = 6–7 mice per group). (l) Pearson's *r* correlation analyses between GFAP^+^ astrogliosis and 6E10^+^ Aβ for total brain plaque area in PBS‐control and GA‐immunized ADtg mice (*n* = 12 mice). Bar graphs indicate mean, standard error of mean (SEM), and individual data points. *p < 0.05, **p < 0.01, ***p < 0.001, ****p < 0.0001 assessed by unpaired Student's *t* test for two‐group comparisons, and a one‐way ANOVA with Tukey's post‐test for three or more groups

To further explore the therapeutic potential of immunomodulation in old, advanced disease stage mice, we performed in‐depth histological analyses on brain tissues from Cohort 1. As outlined in Figure 1b, both retinas and left posterior brain tissues were allocated for biochemical MSD and MS analyses, and additionally, the right brain hemispheres were evaluated by IHC analyses. Histological examination of various AD‐relevant biomarkers, including Aβ‐plaque burden, both vascular and parenchymal deposits, ionized calcium‐binding adaptor molecule 1 (Iba‐1), protein tyrosine phosphatase receptor type C (CD45), glial fibrillary acidic protein (GFAP), and glutamine synthetase (GS), was conducted on brain tissues encompassing the following regions as specified in Figure 3a: hippocampus (Hipp), cingulate cortex/retrosplenial area (Ctx), and entorhinal cortex/piriform area (Ent; Figures 3 and 4; extended data in Figures S4–S8). Representative microscopic images of cortical regions in GA‐immunized versus PBS‐control old ADtg mice revealed lower frequency of large 6E10^+^‐Aβ plaques and surrounding GFAP^+^ astrogliosis in the GA‐immunized group (Figure 3b vs. 3c). Stereological analysis of Aβ‐plaque area verified significant reductions in all analyzed brain regions following GA immunization (Hipp: 40% reduction, *p* = 0.0003; Ctx: 48% reduction, *p* < 0.0001; Ent: 45% reduction, *p* < 0.0001; Total brain: 45% reduction, *p* < 0.0001; by one‐way ANOVA and Sidak's post‐test; Figure 3d). Further, we analyzed vascular 6E10^+^ Aβ deposits in the Ent of GA‐immunized versus PBS‐control old ADtg mice (Figure 3e–f). Vascular Aβ pathology was categorized along a scale of cerebral amyloid angiopathy (CAA; from none = 0 to severe = 4 scores; Figure 3f). Our quantitative evaluation suggests a significant 63% reduction in vascular amyloidosis scores following GA immunization (*p* = 0.0093, by unpaired two‐tailed Student's *t* test; Figure 3e).

To explore Aβ plaque subtypes possibly targeted by GA immunization, we developed a semi‐manual method to measure individual plaque morphology, classified by size, shape, and density, as presented in Figure 3g–h. Our analysis included 6E10^+^‐Aβ perimeter area, length (maximum diameter), and width (minimum diameter) of individual plaques in GA‐immunized versus PBS‐control mice, further identifying plaque subtypes as dense‐core (DC) or non‐DC (Figure 3g–h). Assessment of individual Aβ‐plaque area within the Ent showed a reduction in plaque size of over 20% (*p* = 0.0167, by unpaired two‐tailed *t* test; Figure S4A). Structural classification of Aβ plaques by density revealed a substantial decrease in number and area of DC plaques, along with other size measurements of this plaque subtype (Figure 3i–k; extended data in Figure S4B‐C). GA immunization in these old ADtg mice resulted in a consistently smaller average area, lower quantity, and shortened length of the dense‐core plaques (*p* = 0.0054 with 28% reduction in size, *p* = 0.0010 with 47% reduction in count, and *p* < 0.0029 with 16% reduction in length of DC plaques; Figure 3i–k). These differences did not reach statistical significance for non‐DC diffuse plaques (Figure S4D–F).

Further stratification by plaque size revealed that GA immunization especially modified large plaques (*p* = 0.0331 with 24% reduction in area, *p* = 0.0055 with 25% decrease in width, and *p* = 0.0017 with 28% decrease in length of large plaques; Figure 3l–n; extended data on other plaque types and measurements see Figure S4G–I). Overall, these results may uncover critically beneficial effects of immunomodulation on limiting pre‐existing Aβ deposits at an old age and advanced disease stage.

### 2.5 Immunomodulation curbs microgliosis and astrogliosis with increased recruitment of peripheral myelomonocytes

Neuroinflammatory responses mediated by innate immune cells surrounding Aβ plaques have been implicated in modulating plaque structure and subsequent toxicity in both animal models of AD and human patients (Rasmussen et al., 2017; Vilella et al., 2018; Wang et al., 2016). Of these, microgliosis and reactive astrocytes are established features around Aβ plaque lesion sites (Heneka et al., 2015; Osborn et al., 2016; Rajendran & Paolicelli, 2018; Rodriguez‐Arellano et al., 2016; Wyss‐Coray & Mucke, 2002). Further, the involvement of peripheral immune cells has been shown in both pathological processes (Bradshaw et al., 2013; Ferretti et al., 2016; Gate et al., 2020; Jevtic et al., 2017; Li et al., 2020; Pluvinage & Wyss‐Coray, 2020) and protective clearance of cerebral Aβ, reduction of neuroinflammation, and promotion of tissue regeneration (Bernstein, et al., 2014; Butovsky et al., 2007; Koronyo et al., 2015; Koronyo‐Hamaoui et al., 2009, 2020; Lebson et al., 2010; Li et al., 2020; Rentsendorj et al., 2018; Simard et al., 2006). To assess changes in microgliosis and infiltrating myeloid cell populations, serial brain sections were immunolabeled against Iba1, marker of brain‐resident microglia and peripheral myeloid cells, CD45^hi^ for infiltrating hematopoietic immune cells, and GFAP for astrocytes.

There were substantial increases in cerebral Iba1^+^ microgliosis, with activated‐type cell morphology surrounding plaques, as well as in Iba1^+^CD45^hi^ myeloid cell population in old PBS‐injected ADtg mice when compared to matched naïve WT mice (Figure 4a–b; extended representative images in Figure S5–S7). This was evident throughout the analyzed brain regions, including Hipp, Ctx, Ent, and total brain regions (*p* < 0.0001 for all comparisons, by one‐way ANOVA and Sidak's post‐test; Figure 4a–b). Importantly, even at old age, our immunomodulation strategy considerably diminished Iba1^+^ microgliosis in ADtg mice, with an average reduction of 56% in total brain regions (*p* < 0.0001, by one‐way ANOVA and Sidak's post‐test; Figure 4a). In contrast, immunization further induced recruitment of peripherally derived Iba1^+^CD45^hi^ myeloid cells into the Hipp, Ctx, and Ent regions, by an average 78% increase in total brain in GA‐immunized vs PBS‐control ADtg mice (*p* < 0.001–0.0001; Figure 4b). Colocalization analysis of Iba1^hi^CD45^hi^ out of total Iba1^+^ population revealed that the portion of infiltrating innate immune cells was significantly increased from 27% in PBS‐control mice to 40% in GA‐immunized old ADtg mice (*p* = 0.0061, unpaired Student's *t* test; Figure 4c).

Representative microscopic images of PBS‐control and GA‐immunized old ADtg mouse brains show GFAP^+^ reactive astrocyte and Iba1^+^ myelomonocytic cell populations surrounding 4G8^+^‐Aβ plaques (Figure 4d–f). Observations of inflammatory Iba1^+^ cells found higher numbers per plaque site in GA‐treated as compared to PBS‐control brains and displayed a higher colocalization with Aβ (Figure 4d–f), which may indicate increased Aβ‐plaque uptake. Representative microscopic images in Figure 4g demonstrate the abundance of cortical Iba1^+^CD45^hi^ tagged peripheral immune cells following GA immunomodulation compared to PBS treatment (extended images in Figures S5–S7). Quantification of the ratio between Iba1^+^CD45^hi^ area and 6E10^+^ Aβ‐plaque area revealed a substantial ~3‐fold increase in these infiltrating myelomonocytes per plaque site in the GA‐immunized versus PBS‐control mouse brains (*p* < 0.0001, by unpaired Student's *t* test; Figure 4h).

Correlational analysis revealed a very tight inverse association between CD45^hi^ area and 6E10^+^‐plaque area in Ctx of old ADtg mice (*r* = −0.96 and *p* < 0.0001; Figure 4i; extended Pearson's correlations between either brain‐resident or peripherally derived myeloid cells and plaque area in different AD‐relevant brain regions are in Figure S8A–B). When analyses were separated by experimental group, GA‐treated mice retained a strong correlation between these infiltrating myeloid cells and plaque area, while the PBS‐control group did not display a significant association (*r* = −0.97 and *p* = 0.0016,*r* = −0.54 and *p* = 0.2669, respectively; Figure 4j). These results may indicate that cerebral infiltration of Iba1^+^CD45^hi^ myeloid cells induced by GA immunization is strongly linked with clearance of Aβ pathology.

GFAP is one of the key biomarkers for detecting reactive, scar‐tissue related astrocytes (De Strooper & Karran, 2016; Osborn et al., 2016). Quantitative IHC analysis of GFAP^+^ reactive astrocyte area showed greater cerebral astrogliosis in PBS‐control old ADtg mice as compared to the old wild‐type mice (*p* < 0.0001; Figure 4k). GA immunization consistently curtailed astrocytosis by 30 and 35% in various brain regions, including Hipp, Ctx, Ent, and total brain (*p* < 0.0001, *p* < 0.0001, *p* = 0.0361, and *p* < 0.0001, respectively, by one‐way ANOVA and Tukey's post‐test correction; Figure 4k). Moreover, levels of cerebral GFAP^+^ astrocytosis directly correlated with 6E10^+^‐Aβ burden (Pearson's *r* = 0.84 and *p* = 0.0006; Figure 4l; extended correlation analyses per brain region in Figure S8C).

Next, we evaluated the astrocyte‐specific biomarker glutamine synthetase (GS), an enzyme associated with GFAP reactivity and responsible for synaptic recycling of extracellular glutamate (Rudy et al., 2015; Son et al., 2019; Zou et al., 2011). Peroxidase‐based immunostaining for GS in coronal brain sections demonstrated morphological and intensity differences between GA‐immunized and PBS‐control old ADtg mice (Figure S8D). The GA‐immunized group showed patterns indistinguishable from those of the non‐transgenic WT mice. Across the three experimental groups, GFAP^+^ reactive astrocyte area was associated with levels of GS immunoreactive area (Pearson's *r* = 0.72 and *p* = 0.0005; Figure S8E). Quantitative IHC analysis revealed a significant increase in GS^+^ area in PBS‐control ADtg mice when compared to naïve WT mice (*p* = 0.0244), with levels of GS normalized to WT in the Ent of GA‐immunized ADtg mice (*p* < 0.05; Figure S8F). Overall, our data suggest that GA immunization in old AD model mice has multiple beneficial effects toward reducing Aβ plaques and restoring homeostatic microglia and astrocytic milieu, along with recruitment of Iba1^+^CD45^hi^ myeloid cells.

### 2.6 Synaptic preservation by GA immunization in old ADtg mice

**Figure 5:**
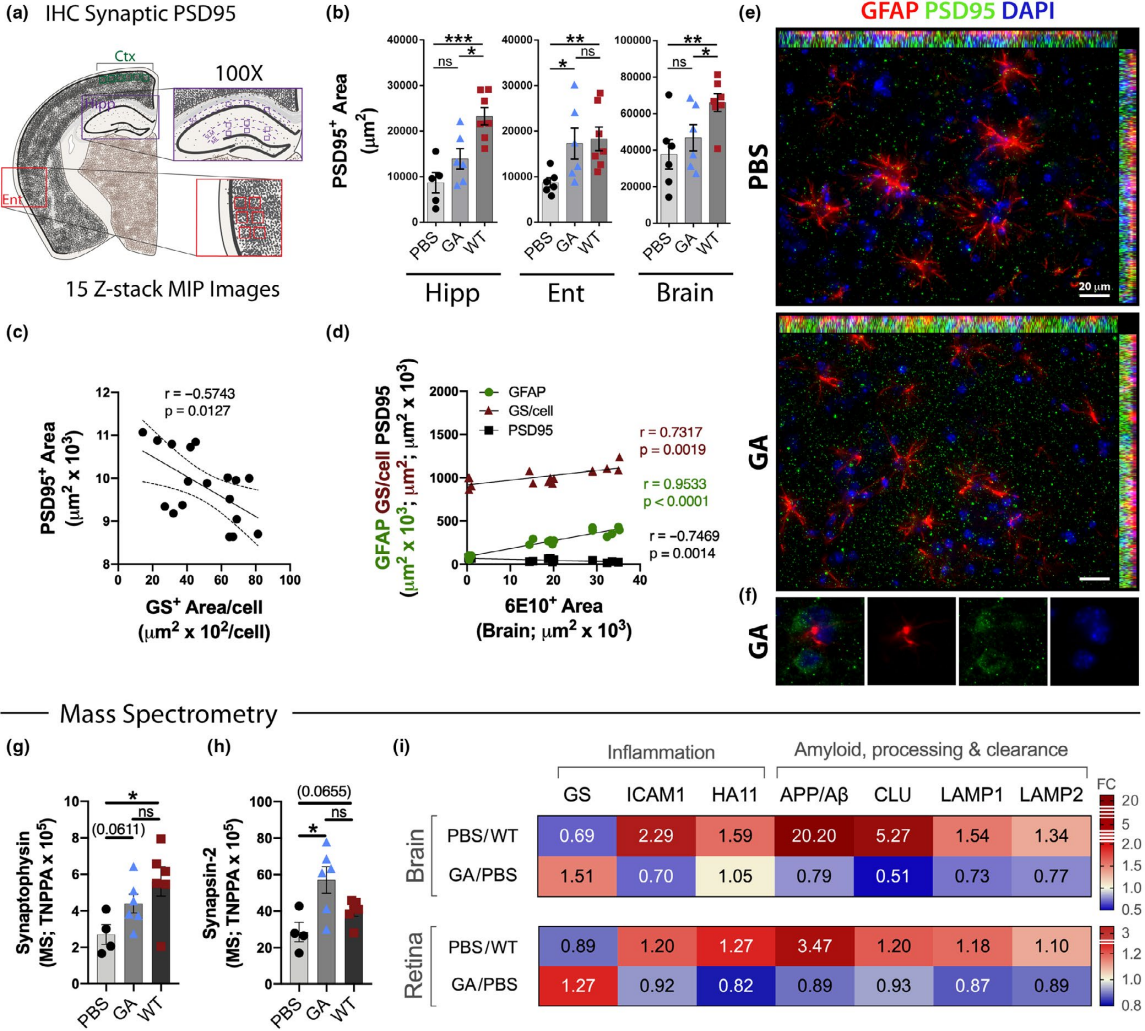
Effects of GA immunization on synaptic density and AD‐related retino‐cerebral proteins in old ADtg mice. (a) Coronal section map displaying specific brain regions analyzed by IHC; regions include the cingulate cortex/retrosplenial area (Ctx), hippocampus (Hipp), and entorhinal cortex/piriform area (Ent). Magnification of 100× microscopic images covering these brain regions was analyzed for post‐synaptic biomarker (PSD95). 15 consecutive *z*‐stack images were captured with ApoTome‐equipped Zeiss microscope provided high‐resolution images of synaptic puncta. (b) Quantitative analysis of PSD95^+^ synaptic area assessed in Hipp, Ent, and combined brain regions in all experimental groups (*n* = 6–7 mice/group). (c) Pearson's *r* correlation analysis between total brain PSD95^+^ synaptic area and astrocytic marker glutamine synthetase (GS)^+^ area per cell (*n* = 18 mice). (d) Collective Pearson's *r* correlation of 6E10^+^ Aβ‐plaque burden against PSD95^+^ post‐synaptic density (black), GS^+^ area/cell (brown), and GFAP^+^ astrogliosis (green) in the Ent (*n* = 10–12 mice). (e) Representative fluorescent images (40×) of coronal brain sections from Ent stained for astrocytes (GFAP, red), post‐synaptic protein (PSD95, green), and cell nuclei (DAPI, blue) from PBS‐control (top) and GA‐immunized (bottom) old ADtg mice. (f) Representative, high‐magnification images (100×) of a GA‐immunized ADtg mouse brain showing density of PSD95 density. (g–h) Mass spectrometry analysis of total normalized peak protein area (TNPPA) of significantly changed synaptic proteins, including (g) synaptophysin and (h) synapsin‐2, between all experimental groups (*n* = 4–6 mice per group). (i) Heat map displaying relative fold change of significantly changed proteins in mass spectrometry analysis. Highlighted are AD‐related amyloid‐associated markers (amyloid‐β A4 protein—APP/Aβ, clusterin—CLN, lysosomal‐associated membrane protein 1 and 2—LAMP1/2) and inflammatory markers (glutamine synthetase—GS, intercellular adhesion molecule 1—ICAM1, h‐2 class I histocompatibility antigen—HA11) that were significantly up‐ or down‐regulated in brain and retinal tissues of PBS‐control ADtg mice versus naïve WT mice and/or GA‐immunized versus PBS‐control ADtg mice (*n* = 4–6 mice per group). Bar graphs indicate mean, standard error of mean (SEM), and individual data points. *p < 0.05, **p < 0.01, ***p < 0.001, by one‐way ANOVA with Tukey's post‐test for three or more experimental groups. Mass spectrometry analysis by unpaired Student's *t* test. Pearson's *r* correlation analysis was used to determine statistical association

The critical role of astrocytes in synaptic homeostasis, notably GS activity in regulating extracellular glutamate, prompted us to evaluate synaptic integrity and its relationship to astrocytic GS expression in these old ADtg mice. Histological examination of synaptic density was assessed for the post‐synaptic density protein 95 (PSD95) biomarker in three AD‐relevant brain regions, including hippocampal subregions, as outlined in Figure 5a. A significant increase in PSD95^+^ area, reflecting improved synaptic density, was seen in the Ent region following GA immunization in ADtg mice, reaching equivalent levels to those measured in old WT littermates (*p* = 0.0411 and 98% increase; Figure 5b; extended analysis of various anatomical hippocampal layers is shown in Figure S9A). Post‐synaptic area was inversely correlated with astrocytic GS expression per cell, which was independent of treatment or genotype group (*r* = −0.57 and *p* = 0.0127; Figure 5c). Combined correlations of brain biomarkers indicate a significant inverse relationship between Aβ pathology and post‐synaptic area (PSD95) and direct associations of Aβ burden with GFAP^+^ reactive astrocytosis and astrocytic GS^+^ area/cell (*r* = −0.75 and *p* = 0.0014, *r* = 0.95 and *p* < 0.0001, *r* = 0.73 and *p* = 0.0019, respectively; Figure 5d). Representative fluorescent micrographs illustrate a substantial increase in PSD95^+^ puncta number along with reduced astrocytosis and homeostatic astrocyte morphology following GA immunomodulation (Figure 5e–f; extended data in Figure S9B–C).

Further, to explore effects of GA immunization on retinal and brain tissues from old, late‐stage ADtg mice, a sensitive and quantitative MS proteomic analysis was applied (Figure 5g–i and Table 1; the complete MS datasets of identified proteins are included in Table S1 for brain and Table S2 for retina). The MS data revealed a reduction of cerebral presynaptic synaptophysin and synapsin‐2 levels in PBS‐control ADtg vs naïve WT mice (*p* = 0.0303 and *p* = 0.0655, respectively, by one‐way ANOVA; Figure 5g–h). Importantly, in accordance with histological findings of postsynapses, a significant increase in levels of synapsin‐2 and a trend of elevation for synaptophysin were observed for GA‐immunized versus PBS‐control ADtg mice (*p* = 0.0112 and *p* = 0.0611, respectively, by one‐way ANOVA; Figure 5g–h).

**Table 1:** Quantitative mass spectrometry analysis of preselected proteins from brain and retinal tissue

	Protein	Abbreviation	WT Ξ (± SEM)	ADtg‐PBS Ξ (± SEM)	ADtg‐GA Ξ (± SEM)	FC ADtg vs. WT	P‐value (*t* test)	FC GA vs. PBS	P‐value (*t* test)
Brain									
Synaptic markers	Synaptophysin	SYPH	5.6 (± 0.8)	2.7 (± 0.5)	4.4 (± 0.5)	0.48	**0.0288**	1.63	0.0611
	Synapsin—2	SYN2	40.0 (± 2.6)	28.6 (± 5.3)	57.1 (± 7.3)	0.72	0.0655	2.00	**0.0218**
Inflammation	Glutamine synthetase	GS	82.6 (± 5.4)	56.8 (± 10.9)	85.5 (± 6.8)	0.69	0.0536	1.51	**0.0390**
	Intercellular Adhesion Molecule 1	ICAM1	0.8 (± 0.1)	1.7 (± 0.2)	1.2 (± 0.2)	2.29	**0.0026**	0.70	0.1800
	H‐2 Class I Histocompatibility Antigen	HA11	0.3 (± 0.02)	0.5 (± 0.1)	0.6 (± 0.04)	1.59	**0.0086**	1.05	0.7174
Amyloid processing and clearance	Amyloid‐β A4 Protein	APP/Aβ	5.4 (± 0.5)	109.6 (± 33.2)	86.1 (± 14.8)	20.20	**<0.0001**	0.79	0.4809
	Clusterin	CLU	13.6 (± 1.9)	71.7 (± 26.2)	36.8 (± 4.4)	5.27	**0.0237**	0.51	0.1423
	Lysosomal‐Associated Membrane Protein 1	LAMP1	1.9 (± 0.2)	2.96 (± 0.3)	2.2 (± 0.1)	1.54	**0.0102**	0.73	**0.0037**
	Lysosomal‐Associated Membrane Protein 2	LAMP2	2.5 (± 0.1)	3.3 (± 0.8)	2.6 (± 0.5)	1.34	**0.0287**	0.77	0.0787
Retina									
Inflammation	Glutamine synthetase	GS	323.3 (± 30.1)	286.3 (± 13.4)	363.7 (± 32.5)	0.89	0.2419	1.27	**0.0001**
	Intercellular Adhesion Molecule 1	ICAM1	1.74 (± 0.1)	2.1 (± 0.1)	1.9 (± 0.1)	1.20	**0.0122**	0.92	0.2492
	H‐2 Class I Histocompatibility Antigen	HA11	0.5 (± 0.03)	0.7 (± 0.04)	0.6 (± 0.1)	1.27	**0.0101**	0.82	0.2914
Amyloid processing and clearance	Amyloid‐β A4 Protein	APP/Aβ	3.1 (± 0.2)	10.9 (± 0.3)	9.6 (± 0.5)	3.47	**<0.0001**	0.89	**0.0396**
	Clusterin	CLU	4.7 (± 0.2)	5.6 (± 0.3)	5.2 (± 0.3)	1.20	**0.0204**	0.93	0.3385
	Lysosomal‐Associated Membrane Protein 1	LAMP1	1.1 (± 0.1)	1.3 (± 0.2)	1.1 (± 0.1)	1.18	0.4068	0.87	0.5359
	Lysosomal‐Associated Membrane Protein 2	LAMP2	7.3 (± 0.3)	8.0 (± 0.3)	7.1 (± 0.1)	1.10	0.0965	0.89	**0.0287**

Data table includes analysis of PBS‐control, GA‐immunized, and WT naïve experimental groups (*n* = 5–7 mice/group). Protein peak areas were normalized to the total protein peak area of the respective sample and subjected to one‐sample *t* tests to compare relative protein peak areas between the respective sample groups. *t* test *p*‐value smaller than 0.05 and fold change ±1.2 was highlighted as differentially expressed proteins. Ξ—Average of total normalized protein peak area (×10^5^).

### 2.7 Cerebral and retinal AD‐related protein profiles in response to immunomodulation

Next, proteins associated with amyloid, processing, clearance, and inflammation that were similarly and significantly down‐ or up‐regulated in brain and retinal tissues from old mice either in disease or following GA immunization, as determined by MS analysis, are highlighted in Figure 5i. Specifically, parallels between brain and retinal proteins are shown by fold changes for the comparisons of PBS‐control ADtg to naïve WT groups and GA‐immunized to PBS‐control ADtg groups (Figure 5i heatmaps; proteins up‐regulated in red and down‐regulated in blue; extended data with the statistical analyses shown in Table 1). Brain MS data revealed elevated GS concentrations in GA‐immunized versus PBS‐control ADtg mice (51% increase and *p* = 0.0390; Table 1), which were remarkably normalized to levels observed in the WT mice. This response in GS astrocytic function following GA was mirrored in the retina, demonstrates another linkage between these two tissues (27% increase and *p* = 0.0001; Table 1). Compared to WT, the immune markers intercellular adhesion molecule 1 (ICAM1), involved in T cell‐APC adhesion, and the mouse MHC molecule H‐2 class 1 histocompatibility complex (HA11) were increased in ADtg mouse brains (129% increase and *p* = 0.0026, 59% increase and *p* = 0.0086, respectively) and retina (20% increase and *p* = 0.0122, 27% increase and *p* = 0.0101, respectively; Figure 5i heatmaps and Table 1). However, the effects of GA did not reach statistical significance in both tissues.

In the context of amyloid processing and clearance, our quantitative MS analysis of cerebral and retinal APP/Aβ further confirmed substantial increases with disease, albeit six times greater in the brain versus the retina (20‐fold vs. 3.5‐fold increases, respectively, *p* < 0.0001; Figure 5i heatmaps and Table 1). Importantly, retinal APP/Aβ protein levels in ADtg mice were significantly reduced by GA treatment (11% and *p* = 0.0396; Table 1). The Aβ‐related clusterin chaperone protein (CLU) showed substantial increases in cerebral concentration in old ADtg vs WT mice (~5‐fold increase and *p* = 0.0237) and to a lesser but significant extent in the retina (20% increase and *p* = 0.0204). Finally, two lysosomal‐associated membrane proteins 1 and 2 (LAMP1/2) were similarly but to a lesser extent affected by disease and GA immunization in the retina versus the brain. LAMP1 and LAMP2 were significantly elevated in ADtg versus WT mouse brains (54% increase and *p* = 0.0102, 34% increase and *p* = 0.0287, respectively), but did not reach statistical significance in the retina (*p* = 0.4068 and *p* = 0.0965, respectively). Interestingly, GA immunomodulation primarily affected LAMP1 in the brain (27% decrease and *p* = 0.0037) and LAMP2 in the retina (11% decrease and *p* = 0.0287).

## 3 DISCUSSION

This study provides the first evaluation of amyloidogenic and non‐amyloidogenic Aβ peptide levels and Aβ_42/40_ ratios in paired retina and brain tissues from two cohorts of old, late‐stage ADtg mice, including in response to immunomodulation. Our findings demonstrated the potential of the neurosensory retina to predict cerebral Aβ accumulation in disease and following intervention in old ADtg mice, a more relevant model for the age‐dependent human disease. Therapeutic effects of GA immunization in old age were revealed on both retinal and cerebral tissues. Further, we identified lateralization of Aβ_42_, Aβ_40_, Aβ_38_ levels and Aβ_42/40_ ratios between OS and OD retinae and between left and right brain hemispheres. While significant disparities in accumulation of Aβ alloforms between left and right sides of these CNS tissues are detected, levels of Aβ in one side consistently correlated with Aβ levels in the respective side. Moreover, Aβ_40_ burden strongly predicted Aβ_42_ burden in each retina or brain tissue. Furthermore, our data found therapeutic effects of GA immunomodulation at advanced disease stages, as indicated by: (1) cerebral Aβ‐plaque reduction, especially targeting large and dense‐core parenchymal deposits; (2) decreased cerebral vascular amyloidosis; (3) reduced Aβ_42_/Aβ_40_ ratio in the OD retina; (4) attenuation of cerebral GFAP^+^‐astrocytosis and Iba1^+^‐microgliosis; (5) enhanced cerebral Iba1^hi^/CD45^hi^ myelomonocytic population surrounding Aβ plaque; (6) preservation of synaptic integrity; and (7) restoration to homeostatic levels of several retinal and cerebral proteins related to astrocyte function, inflammation, and Aβ processing and clearance, identified by MS proteomics profiling. Collectively, the multifaceted neuroprotection provided by GA immunization in old AD model mice suggests that CNS repair is possible via immunomodulation, even after severe tissue damage occurs. Further, the tight relationship between retinal and brain amyloidogenic Aβ burden in response to immune‐based treatment encourages future exploration of the retina as an advantageous AD biomarker for direct, noninvasive imaging to assess therapeutic efficacy.

The long‐standing view of AD as a disease restricted to the brain has recently been challenged by mounting evidence supporting the manifestation of AD pathological hallmarks in the retina of animal models [reviewed in (Hart et al., 2016; Kusne et al., 2017; Mahajan & Votruba, 2017)] and human patients (Alexandrov et al., 2011; den Haan et al., 2018; Hadoux et al., 2019; Koronyo et al., 2017; Koronyo‐Hamaoui et al., 2011; La Morgia et al., 2016; Schon et al., 2012; Schultz et al., 2020; Shi et al., 2020; Tsai et al., 2014). While the retina is an appealing and accessible CNS organ for live imaging, the tissue's delicate and thin structure along with its small proportion in murine models raises particular technical challenges for ex vivo histological and biochemical assays. To overcome this hurdle, we developed an experimental protocol that allowed for quantitative measurements of neurosensory retinal peptides and proteins, including those with highly aggregative, amyloidogenic tendencies. This technique involved the separation of the neuro‐retina from freshly dissected and snap‐frozen eyes, concentration of tissue proteins, and subsequent denaturation of aggregates with epitope exposure by chemical conformational unraveling. Application of this method allowed for highly sensitive and quantitative triple‐Aβ alloform analyses by MSD assays.

Our study revealed for the first time an unexpected lateralization in Aβ concentrations in OS versus OD retinae for Aβ_42_, Aβ_40_, and Aβ_38_ alloforms, in addition to AD‐relevant Aβ_42_/Aβ_40_ ratio levels. Individual Aβ alloforms were consistently and substantially higher in the OS compared with OD retina, with higher Aβ_42_/Aβ_40_ ratios in the OD retina, indicating lateralization of Aβ levels in the retina of these old, advanced‐stage ADtg mice. Similarly, a previous study in AD human retina demonstrated lateralization in structural pathology, including RNFL thinning and tissue degeneration, with more pronounced pathology in the OS‐left versus OD‐right retina (Hwang et al., 2014). Importantly, our results of left posterior brain‐dominant lateralization of Aβ_42/40_ ratio is in agreement with previous studies in AD patients that identified left greater than right disease‐associated cortical and white matter atrophy (Long et al., 2013; Mesulam et al., 2014; Minkova et al., 2017; Wahlund et al., 1993). In addition, AD patients have been reported to display reductions in left hemisphere glucose metabolism (Lehmann et al., 2013; Toga & Thompson, 2003; Weise et al., 2018), which has been associated with functional declines in verbal fluency (Weise et al., 2018). Other structural and molecular measurements also indicated lateralization between the two hemispheres, including a number of studies showing left‐dominant accumulation of tauopathy, which could even predict disease onset (Mesulam et al., 2014; Minkova et al., 2017; Tetzloff et al., 2018; Wachinger et al., 2016). Most interestingly, in patients with right‐hand dominance there was higher left‐side brain tauopathy, while in patients with left‐hand dominance and known right‐hemisphere language dominance, tau burden lateralization shifted to the right hemisphere (Mesulam et al., 2014). Hence, it is postulated here that our findings of side‐dominant susceptibility of Aβ pathogenesis could be an outcome of differences in eye dominance, neuronal firing and circuit activity, and metabolism, eventually affecting Aβ production, processing, propagation, or clearance processes.

Nevertheless, despite these disparities, there was a close linear correlation in levels of all Aβ alloforms between the left and right retinae. In addition to having higher Aβ peptide concentrations, the OS retina appears to better predict as compared to the OD retina its cerebral counterpart. If this disparity holds true in human patients, this may have implications in research methodologies and even organ acquisition. Additionally, results from prior investigations on retinal and brain pathology, averaging data from both eyes and/or brain hemispheres, may be skewed. Future studies are warranted to establish retinal and brain Aβ‐burden lateralization and investigate the possible explanation of such lateralization in order to better understand processes regulating CNS Aβ accumulation and removal.

While two previous reports have analyzed ocular Aβ burden by ELISA in several transgenic murine AD models (Alexandrov et al., 2011; Parthasarathy et al., 2015; Schultz et al., 2020), they did not include analysis of paired retinas and brains, lateralization assessment, or they did not separate the neurosensory retina from other ocular tissues. Our previous investigations identified for the first time the presence of Aβ plaques and characterized their morphology in retinas from AD patients and early‐stage cases (Koronyo et al., 2017; Koronyo‐Hamaoui et al., 2011; La Morgia et al., 2016; Shi et al., 2020). Moreover, our preliminary data detected a correlation between paired retinal Aβ_42_‐containing plaques and cerebral plaque burden in these patients (Koronyo et al., 2017). In terms of retinal and brain response to therapy, reports in 12‐ and 14‐month‐old murine models of AD showed similar reductions in retinal and brain Aβ plaques following immunization with dendritic cells loaded with MOG45D (Koronyo‐Hamaoui et al., 2011) or with anti‐Aβ antibodies (Hwang et al., 2014; Liu et al., 2009). However, this was never precisely quantified by biochemical methods, nor measured specifically in old mice following GA immunization. Importantly, the feasibility to detect in vivo individual Aβ plaque appearance and clearance dynamics in real time via a noninvasive retinal curcumin optical imaging method (Koronyo et al., 2012), and with aging (Sidiqi et al., 2020), encourages utilization of retinal amyloid imaging to gauge response to therapy with high spatial resolution. One limitation of this study is the lack of live retinal/brain imaging data to correlate with the histopathological findings. Our results rely on histological and biochemical analyses, revealing new phenomena that support the need to further correlate between live retinal/brain amyloid imaging and AD‐related pathological biomarkers. Future investigation using live retinal imaging is warranted to identify such AD biomarkers, especially to facilitate evaluation of response to therapy.

The current studies led us to reveal the following fundamental unknowns regarding the potential connection between retinal and brain pathology in ADtg mice: (1) While the levels of retinal Aβ_38_, the more benign alloform, weakly corresponded to its left‐posterior brain counterpart, the levels of retinal Aβ_40_ and Aβ_42_, the synaptotoxic and AD‐pathognomonic alloforms (Raskatov, 2019), tightly predicted the levels of their brain counterparts, especially following GA immunization, (2) As mentioned above, there were consistent disparities in accumulation of all Aβ alloforms in left versus right retina and left versus right brain hemispheres, and (3) The therapeutic effects of GA immunomodulation were established at late‐stage disease in the old ADtg mice, including notable parallels in molecular markers between retinal and cerebral tissues in disease and with therapy beyond Aβ burden.

While non‐significant small reductions were detected in cerebral Aβ alloforms following GA immunization in old ADtg mice, GA did lead to substantial decreases in cerebral vascular and parenchymal Aβ plaques along with significant mitigations of microgliosis, astrocytosis, along with induction of homeostatic astrocyte profile. The measurement of Aβ alloforms represents concentrations of both soluble and insoluble Aβ forms, including the monomers, dimers, tetramers, oligomers, and other intermediates, which is beyond the plaques, and can explain the discrepancy between the histological and biochemical data. Moreover, the results of induced cerebral myeloid phagocytic cell recruitment and homing to the plaques sites may explain increased plaque clearance and synaptic preservation. Although one limitation of this study is the lack of cognitive testing, which was due to enhanced frailty and attrition rate among these old ADtg mice, our results of pre‐ and post‐synaptic preservation (e.g., PSD95, synaptophysin, synapsin‐2), which are typically predictive of cognitive function (Ferreira et al., 2015; Shankar et al., 2008), may indeed reflect a functional preservation.

Interestingly, the strength of the association displayed here between the OS and OD retina in levels of pathognomonic Aβ_42_ was very similar to those observed between the retinae (individually and together) and their cerebral counterparts. Moreover, we found in our old ADtg mouse cohorts that the ratio between Aβ_42_ and Aβ_40_, which is another key measure of AD neuropathology (Rembach et al., 2014; Schindler et al., 2019), was tightly regulated and similar across brain and retinal tissues. Importantly, the only imbalance in Aβ_42/40_ ratio that was found in the OD retina of old ADtg mice was restored to levels equal to other measured CNS tissues by GA. The Aβ_42/40_ ratio is thought to more accurately depict pathological burden and has been indicated in elevated protein toxicity when compared to single alloform levels alone (Kuperstein et al., 2010). This phenomenon may manifest as an OD‐dominant lateralization in retinal and left‐brain degenerative pathologies, which should be studied in future investigations. This result may also entail that despite differences in levels, the relative rates of Aβ_42_ and Aβ_40_ accumulations, affected by production and clearance, either in the retina or brain, are tightly connected, and potentially gives insight into another aspect of GA therapeutic impact.

In this study, we found that the concentrations of each Aβ alloform are substantially lower in the retina than in the brain, which is in accordance with previously published results in animal models and human donors (Alexandrov et al., 2011; Grimaldi et al., 2018; Koronyo et al., 2017; Koronyo‐Hamaoui et al., 2011; Schultz et al., 2020; Shi et al., 2020). Of note, it is believed that the concentrations of Aβ_42_ deemed detrimental or toxic to brain cells and can lead to LTP deficits are dose‐dependent (Raskatov, 2019). Whether and how retinal concentrations translate to retinal cell toxicity and pathology warrant future investigation. Nonetheless, growing histological evidence, both in humans and animal models, shows that retinal Aβ depositions are tightly associated with local neuronal dysfunction (ERG), RNFL loss, RGC degeneration, and general tissue atrophy (Asanad et al., 2019; Grimaldi et al., 2018; Hart et al., 2016; Huang et al., 2017; Ju et al., 2013; Koronyo et al., 2012, 2017; La Morgia et al., 2016; Lei et al., 2017; Ning et al., 2008). Future studies should establish the detrimental retinal Aβ concentrations, assemblies, and topographical locations in AD.

Early retinal and cerebral vascular dysfunctions have been tightly correlated with cognitive impairments related to AD (Attems & Jellinger, 2004; Brenowitz et al., 2015; DeCarli et al., 2019; Liesz, 2019; O’Bryhim et al., 2018; Querques et al., 2019; Shi et al., 2020; Thal et al., 2008). Our findings of substantial reduction of vascular entorhinal cortex deposits in the old GA‐immunized ADtg mice should be clinically relevant since approximately 85 and 90% of AD patients also present with CAA (Brenowitz et al., 2015; Thal et al., 2002, 2008). The latter may be the result of deficiencies in Aβ clearance through the blood–brain barrier (BBB; Bourassa et al., 2019; Hecht et al., 2018). Recent studies in human patients and animal models have demonstrated that amyloid deposition in vessels reduced blood flow and was associated with various other BBB and blood‐retina barrier (BRB) dysfunctions, PDGFRβ down‐regulation, as well as pericyte loss (Deane et al., 2009; Kimbrough et al., 2015; Nation et al., 2019; Ramanathan et al., 2015; Schultz et al., 2018; Shi et al., 2020; Sweeney et al., 2018; Zlokovic, 2011). In addition, astrocytes and their projections, called astrocytic end feet, extend to the walls of blood vessels and are considered a pivotal component of the neurovascular unit (Liu et al., 2018; Verkhratsky & Nedergaard, 2018). Our data indicate that immunomodulation was able to substantially curb reactive astrocytosis even at such an advanced disease stage, conceivably having a positive effect on BBB integrity and Aβ clearance. Notably, our measurements of astrocytic phenotype directly and strongly correlated with severity of vascular and parenchymal Aβ pathology. Future studies should look more closely into vascular dysfunction in AD and specifically assess the integrity of BBB/BRB‐related biomarkers including astrocytic end feet (e.g., aquaporin‐4), to investigate potential roles in BBB/BRB and lymphatic/glymphatic Aβ clearance processes.

Results from the current study, together with several previous reports in younger adult ADtg mice, establish the therapeutic prospective of GA immunization in preclinical models of AD (Bakalash et al., 2011; Baruch et al., 2015; Butovsky et al., 2006, 2007; Frenkel et al., 2005; Koronyo et al., 2015; Li et al., 2020; Rentsendorj et al., 2018). The combined evidence for curbing cerebral Aβ‐plaque burden led us to undertake in‐depth analysis of plaque subtypes to determine which type is primarily targeted by this immunomodulatory approach. Our morphological assessment of Ent Aβ deposits suggested that GA immunization in old mice was most effective in reducing the size and number of large and dense‐core plaques. These plaque subtypes were previously implicated in triggering neuroinflammation and increased neurotoxicity (Busche et al., 2008; Koffie et al., 2009; Li et al., 2020; Rasmussen et al., 2017; Selkoe, 2008; Shankar et al., 2008; Wang et al., 2016). In this regard, peripherally derived myelomonocytes were shown to more effectively recognize and phagocytose plaques comprised of fibrillar Aβ forms versus soluble oligomers (Bernstein, et al., 2014; El Khoury et al., 2007; Koronyo et al., 2015; Lebson et al., 2010; Michaud et al., 2013; Rentsendorj et al., 2018; Zuroff et al., 2017). Nonetheless, GA activation of macrophages also induced a more effective extracellular degradation and clearance of soluble and oligomeric Aβ_42_ forms, thereby protecting neurite structures and synaptic density (Koronyo et al., 2015; Li et al., 2020). This effect on oligomers was not specifically assessed here. Our histological observations, however, highlighted that in brains of GA‐immunized mice, there was a spatial organization of astrocytes and microglia/macrophages surrounding Aβ plaques that may enhance the physical barrier, better protect neuronal network, and improve Aβ clearance. The organized appearance of GFAP^+^ astrocytes is most probably due to less activated astrocytes in between plaques. Future studies of the possible effects of GA immunotherapy on brain and retinal oligomeric Aβ burden and associated gliosis in old ADtg mice are warranted.

Among the novel molecular mechanisms of GA immunization identified here, we found that levels of brain and retinal glutamine synthetase were normalized to levels comparable to those found in healthy WT mice via immunohistochemistry and mass spectrometry. Astrocyte‐associated GS plays a crucial role in the balance of extracellular‐synaptic glutamate levels, converting its excess into glutamine, and is integral in the well‐established tripartite synapse model [reviewed in (Liu et al., 2018; Rudy et al., 2015)] (Allen & Barres, 2009; Allen & Eroglu, 2017; Belanger et al., 2011; Danbolt, 2001). Hence, GA‐induced restoration of GS physiological levels may explain reduced excitotoxicity and preserved synapses (Ortinski et al., 2010; Son et al., 2019; Tani et al., 2014). In accordance with our MS data, dysfunctions in glutamate recycling processes were implicated in AD and drastic declines in the excitatory amino acid transporter 1 (EAAT1) of astrocytes within human tissue and astrocytic cultures exposed to Aβ oligomers have been reported (Huang et al., 2018; Zoia et al., 2004). Interestingly, GS immunoreactive area adjacent to plaques tightly associated with post‐synaptic density, suggesting that restoration of GS expression to homeostatic WT levels in Aβ‐burdened brain regions could prevent synaptic loss. The re‐establishment of resting astrocyte morphology by GA identified by GFAP and GS histological IHC patterns and MS analysis further supports therapeutic efficacy at advanced disease stage.

Our quantitative MS analysis also showed that both retinal and cerebral tissues in old ADtg mice displayed significant up‐regulation of several inflammatory‐related proteins such as ICAM1, an adhesion molecule found on antigen‐presenting cells and pericytes which facilitates transport of innate immune cells across the BBB (Martens et al., 2020; Proebstl et al., 2012). These findings are in line with previous studies reporting up‐regulation of cerebral ICAM1 in a number of AD transgenic models (Ferretti et al., 2016) and, moreover, correlations with Aβ and tau pathology in human AD patients (Walker et al., 2017). Increased h‐2 class I histocompatibility antigen (HA11) mouse MHC molecule further implies heightened immune signaling and involvement in retinas and brains of old AD model mice.

A number of markers for amyloid production and cellular processing were markedly increased in the old, late‐stage ADtg mice. Clusterin (CLU; also named Apolipoprotein J) and amyloid‐β A4 protein, encompassing pre‐processed APP and the various Aβ alloforms, showed extensive increases in diseased brains and retinas, corroborating our MSD and IHC analyses of Aβ. Consistent with previous results, the brain displayed a substantial increase in magnitude changes as compared to the retina (Koronyo et al., 2017; Koronyo‐Hamaoui et al., 2011). Finally, the lysosomal‐associated membrane protein 1/2 (LAMP1/2) molecules have also been implicated in the degradation of Aβ fibrils (Barrachina et al., 2006; Gurney et al., 2018) and were found to be up‐regulated in brain and retinal tissues from old ADtg mice. In response to GA immunomodulation, we revealed a preferential down‐regulation of LAMP1 in the brain and LAMP2 in the retina. Overall, GA restored these molecules to levels similar to those observed in the healthy WT mice.

The results from this study substantiate the versatile therapeutic effects of GA immunomodulation on cerebral and retinal tissues in advanced disease stage in old murine models of AD, a more clinically relevant animal model. A successful immunomodulation approach at such late‐stage disease supports future exploration of such an immunomodulatory strategy for AD treatment. Importantly, the concentrations of AD‐associated Aβ_40_ and Aβ_42_ in the retina predicted their levels in the brain, especially following GA immunization. The intriguing retinal and cerebral lateralization of Aβ_42_, Aβ_40_, and Aβ_38_ alloforms and Aβ_42/40_ in these old mice may lead to reveal the environmental conditions that impose greater susceptibility or protection to AD. Altogether, the correlation of AD‐related pathology and response to therapy of the retina and brain encourages future development of noninvasive retinal biomarkers to detect and monitor therapeutic efficacy.

## 4 EXPERIMENTAL PROCEDURES

### 4.1 Mouse model and experimental groups

Double‐transgenic mouse models for Alzheimer's disease from the B6.Cg‐Tg (APP_SWE_, PS1_∆E9_) 85Dbo/Mmjax strain (Jackson Laboratory, MMRRC Stock #34832‐JAX; ADtg, *n* = 14) and their age‐ and gender‐matched non‐transgenic (WT, *n* = 7) littermates (Cohort 1). Animals were aged until 20 months and then divided into three experimental groups in equal numbers (*n* = 7, 3F/4 M): naïve wild‐type (WT), PBS‐injected ADtg control and GA‐immunized ADtg mice (Figure 1a–b). An additional cohort of double‐transgenic mouse models for AD from the B6.Cg‐Tg (APP_SWE_, PS1_∆E9_) 85Dbo/Mmjax strain (Jackson Laboratory, MMRRC Stock #34832‐JAX; ADtg, *n* = 15) were aged until an average age of 18 months (Cohort 2; 2F/13 M; Figure 1c). In general, this murine model displays an AD‐like neuropathology and symptoms. Such neuropathies and behavioral changes include vast parenchymal and vascular Aβ accumulation, both in its soluble and insoluble forms, astrocytic, microglial, and molecular inflammatory responses, in addition to synaptic loss and subsequent cognitive decline (Jankowsky et al., 2004; Meyer‐Luehmann et al., 2008). This study was performed according to regulations of the Cedars‐Sinai Medical Center Institutional Animal Care and Use Committee under an approved protocol.

### 4.2 GA immunization and tissue allocation

ADtg mice from Cohort 1 were subcutaneously injected with 200 μl of either PBS or 100 μg glatiramer acetate (GA; also known as Copaxone^®^, TEVA Neuroscience) in PBS. Injections were administered twice a week for the first 2 weeks, then once a week for the following 6 weeks—totaling 10 injections. A week after the completion of treatment period, WT and ADtg mice reached an average age of 22.4 months; they were euthanized with perfusion using ice‐cold saline supplemented with 0.5 mM EDTA, as previously described (Koronyo et al., 2015). Brains right hemisphere were collected and fixed overnight in 2.5% paraformaldehyde (Sigma‐Aldrich), then cryo‐protected in 30% sucrose. The entirety of the right brain hemisphere was separated for immunohistochemistry analyses. The left posterior brain hemisphere (divided at Bregma −1 mm) was snap frozen for protein analyses and stored at −80°C. Eyes, Oculus Sinister (OS) and Oculus Dexter (OD), were flash‐frozen in dry ice then stored at −80°C until retinal extraction and protein analyses. The experimental design and allocation of tissue for Cohort 1 are detailed in Figure 1a–b. At the average age of 18 month, Cohort 2 was euthanized and perfused under the same protocol described above. The left and right posterior brains, as well as both OS and OD retinae, were separated for protein analyses. All experiments were conducted and recorded by researchers blinded to the mouse genotypes and the treatment group. The experimental design and allocation of tissue for Cohort 2 are detailed in Figure 1c.

### 4.3 Retinal and brain protein isolation and processing for meso scale discovery (MSD)

Frozen mice eyeballs were placed in a cold solution of 1% Protease Inhibitor in PBS whereupon the cornea and iris were separated, and lens extracted along with the vitreous, being careful not to damage the retina. Using a pair of forceps, the retina was carefully detached from the sclera and snipped at the base of the optic nerve. The retinal weight was recorded and tissue subsequently frozen on dry ice. Posterior brain tissues were separated, weight was recorded, and frozen on dry ice. A lysis buffer containing 0.15 M NaCl, 1 mM Ethylenediaminetetraacetic acid/EDTA, 1% Triton X, and 1% Protease Inhibitor was added to each retina and brain tissue and homogenized via rod sonication (4 Hz; for two cycles of 1.5 min in 15‐s increments). The homogenized tissue, including soluble and insoluble fractions, was then placed into a Speed Vac Concentrator for 3.5 h. The remaining lyophilized tissue pellet was resuspended in a solution of >99% hexafluoroisopropanol (HFIP; Sigma), then homogenized via rod sonication (4 Hz; for 1 cycles of 1.5 min in 15‐s increments) and left overnight (12 h) unraveling the aggregated amyloidogenic proteins for more accurate and sensitive detection. After evaporation of the HFIP, the pellet was resuspended in sterile PBS following homogenized via rod sonication (4 Hz for 1 cycles of 1.5 min in 15‐s increments) and used to determine the protein levels of Aβ_42_, Aβ_40,_ and Aβ_38_ by Meso Scale Discovery (MSD; #K15199G‐1). A schematic illustration of this protocol is shown in Figure 1d. For data regarding both retinae, an average of the OS and the OD retinal values was obtained and designated as “Both” for each mouse individually.

### 4.4 Immunohistochemistry (IHC)

Mouse right brain hemispheres from Cohort 1 were coronally sectioned into 30‐μm thick slices and stored in a 0.01% solution of sodium azide (NaN_3_) in PBS. Brain sections were washed in PBS then incubated in blocking buffer (Dako #X0909), followed by primary antibody incubation overnight in 4°C with combinations of the following primary antibodies in 10% blocking buffer (3–6 sections/mouse): mouse anti‐human Aβ [residues 1–16, mAb clone 6E10 (1:200; #803001; BioLegend), and residues 17–24, mAb clone 4G8 (1:200; #800701; BioLegend)], rabbit anti‐glial fibrillary acidic protein (GFAP) pAb (1:100; G9269; Sigma‐Aldrich), rat anti‐GFAP mAb (1:1,000; #13‐0300; Thermo Fisher), mouse anti‐Glutamine Synthetase mAb (1:200; SC‐74430; Santa Cruz Biotechnology), rabbit anti‐PSD95 mAb (1:600; ab76115; Abcam), guinea pig anti‐VGluT1 pAb (1:6,000; AB5905; Millipore), goat anti‐Iba1 pAb (1:500; NB100‐1028; NovusBio), rabbit anti‐Iba1 pAb (1:200; #019‐19741, Wako Chemicals USA), rat anti‐CD45 mAb (1:25; #550539; BD PharMingen). Sections were then washed in PBS before incubation at 37°C for 1 h with secondary polyclonal antibodies (donkey anti‐mouse, anti‐rat, anti‐goat, anti‐guinea pig, and anti‐rabbit; 1:200; Jackson ImmunoResearch Laboratories) conjugated with Cy2, Cy3, Cy5, or DyLight™ 649. Sections were finally washed in PBS then mounted using ProLong^®^ Gold antifade reagent with DAPI (Molecular Probes, Life Technologies). Routine negative controls for IHC assessments were processed using the same immunolabeling protocol with the omission of the primary antibody to assess non‐specific signal. “Serial brain sections” are defined as separated by five slices. Images were repeatedly captured at the same focal planes with the same exposure time using a Carl Zeiss Axio Imager Z1 fluorescence microscope (Carl Zeiss MicroImaging, Inc.) equipped with ApoTome, AxioCam MRm, and AxioCam HRc cameras. Images were captured at 20 ×, 40 ×, 63 ×, and 100 × objectives for different purposes.

### 4.5 GS mouse‐on‐mouse peroxidase staining

For mouse primary GS anti‐mouse antibodies, a Vector^®^ M.O.M.™ (mouse‐on‐mouse) immunodetection kit (BMK‐2202, Vector Laboratories) was used to reduce non‐specific auto‐reactivity of endogenous proteins. Briefly, sections were incubated in 3% hydrogen peroxide for 10 min at room temperature in order to block endogenous peroxidase activity (3 sections/mouse). A M.O.M IgG Blocking Reagent was applied for 1 h at room temperature. Afterward, sections were left in M.O.M. Diluent for 5 min. GS anti‐mouse primary antibody was diluted (1:200) in M.O.M. Diluent solution and incubated with tissue for 30 min at room temperature. Anti‐mouse IgG M.O.M. Biotinylated reagent in M.O.M. Diluent was applied at room temperature for 10 min before a Vectastain ABC reagent (Vector, PK‐6102, Peroxidase Mouse IgG) was applied. Subsequently, 3,3′‐diaminobenzidine (DAB) in chromogen solution (Dako K3468) was added for visual detection by nonfluorescent immunoperoxidase.

### 4.6 CAA scoring

For amyloid burden assessment in mouse brain vasculature, sections were stained with 6E10 (1:200; #803001; BioLegend) according to a previously described standard protocol (Koronyo‐Hamaoui et al., 2020; Rentsendorj et al., 2018). Various degrees of cerebral amyloid angiopathy (CAA) in animals were defined by analyzing 6E10‐labeled brain sections using a scale of 0–4 (0 indicates no CAA, 4 indicates severe CAA, detailed in Figure 3f; three brain sections per animal), as previously described (Wyss‐Coray & Mucke, 2002). We scored three sections (~10 vessels per image) per animal spanning a 2.25 mm^2^ area of the entorhinal cortex per mouse. These scores were averaged per mouse and compared between PBS‐control and GA‐treated groups.

### 4.7 Amyloid plaque size determination

The number and area (µm^2^) of 6E10^+^ Aβ plaques were determined by examining three coronal sections per mouse spanning the entorhinal cortex region. Five images were taken per section at 20× magnification, and each image was analyzed using Carl Zeiss AxioVision LE software (Carl Zeiss MicroImaging, Inc.). 6E10^+^ Aβ plaques were individually traced using the “outline” and “length” tool for measurements of area, length (longest diameter), and width (shortest diameter). Each plaque was morphologically assessed as either a Dense‐Core plaque, if its core was intensely labeled and clear, or a Non‐Dense‐Core plaque. After analysis of all mouse brain sections, the criteria for Large, Medium, Small, and X‐Small plaques were defined by combining the range of measurements obtained in each category (area: 24.13–11,509.33 µm^2^, length: 5.64–143.5 µm, and width: 5.56–95.66 µm) and observational assessment per image. The criteria were as follows: large area = >1,000 µm^2^, length = >50 µm, width = >30 µm; medium area = 400–1,000 µm^2^, length = 30–50 µm, width = 20–30 µm; small area = 100–400 µm^2^, length = 15–30 µm, width = 10–20 µm; extra‐small area = <100 µm^2^, length = <15 µm, width = <10 µm (detailed in Figure 3h). We covered a total of 2.25 mm^2^ area per mouse brain, and mean values were calculated as the average of 15 images, each spanning 1.5 × 105 μm^2^ area. Analyzers were blinded to the mouse groups when performing all counts.

### 4.8 Synaptic quantification

For synaptic analysis and to cover the entorhinal cortex/piriform area, three of the same rectangular fields (90 μm × 70 μm) under 100× oil objective lens were precisely selected in the lateral and medial blade molecular layer (ML) of the dentate gyrus (DG), the stratum lacunosum‐moleculare (SLM), the stratum radium (SR), and the stratum oriens (SO) of cornu ammonis 1 (CA1) in each condition, respectively. In addition, three of the same fields were carefully chosen in layers 2 and 3 of the entorhinal cortex. Fifteen optical sections per field, nine fields per hippocampal area, six fields per entorhinal cortex/piriform area, five fields per cingulate cortex/retrosplenial area per section, and 300 total images per brain were analyzed. Single optical section images at 0.25 μm intervals and 3.75 μm Zeiss ApoTome high‐resolution scans were performed. Synaptic puncta number and synaptic immunoreactive (IR) area were quantified using Puncta Analyzer 2, 3, and ImageJ (NIH) macro and batch process. Average synaptic area was calculated for each condition.

### 4.9 Sample preparation for mass spectrometry analysis

The protein lysates from brains and retinae were subjected to detergent removal process using detergent removal spin column (Pierce™, Thermo Scientific) as per manufacturer's instructions. Post‐detergent removal, protein amounts were quantified using BCA assay (Pierce™, Thermo Scientific) as per manufacturer's instructions and an equal amount of (50 µg) proteins per samples were subjected to reduction with 10 mM dithiothreitol (DTT) followed by alkylation with 20 mM iodoacetamide (IAA) in the dark. Finally, the reaction was quenched with excess DTT for 15 min. Proteins were digested at 37°C overnight with trypsin at a 1:50 ratio (enzyme to protein ratio). The digests were quenched with formic acid, and peptides were desalted using self‐packed SDB‐RP StageTips (Empore SPE disks) and dried in vacuum centrifuge. Peptide concentration was determined using peptide BCA assay kit as per manufacturer's instructions.

### 4.10 Peptide ion library generation

To produce a peptide ion library of relevant proteins, a pool of peptides was prepared individually for brain and retina sample peptides by mixing equal amounts of respective samples. This identified a base list of proteins from which a subset was chosen that were related to previous IHC analyses. Samples were desalted with C18 Sep Pack Light Cartridges (Waters, USA) and dried down using vacuum centrifugation. Peptides were reconstituted with 5 mM ammonia solution (pH 10.5) and loaded onto an Agilent 300 Extend C18 column (2.1 mm × 150 mm, 3.5 μm, 300 Å). Using a 1,260 quaternary HPLC system, peptides were separated using a linear gradient of 5 mM ammonia solution with 90% acetonitrile (pH 10.5) starting from 3 to 30% for 55 min at a flow rate of 300 μl/min. Peptides were separated into a total of 90 fractions that were consolidated into 17 for liquid chromatography tandem mass spectrometry (IDA‐LC–MS/MS) analysis.

### 4.11 Information‐dependent acquisition mass spectrometry (IDA‐MS)

A 6,600 TripleTOF mass spectrometer (Sciex) coupled to an Eksigent Ultra‐nano‐LC‐1D system (Eksigent, Sciex) was employed for both IDA and SWATH‐MS analysis. HpH fractioned peptide was subjected to 1D‐IDA nano‐LC MS/MS analysis (IDA‐LC–MS/MS) as follows. Each sample was injected onto a reverse‐phase trap for pre‐concentration. The peptide trap (solid core Halo‐C18, 160 Å, 2.7 µm, 100 µm × 3.5 cm) was then switched into line with the analytical column (solid core Halo‐C18, 160 Å, 2.7 µm, 200 µm × 20 cm). Peptides were eluted from the column using a linear solvent gradient of 2 and 30% of mobile phase B over 88 min at a flow rate of 600 nl/min. The reverse‐phase nano‐LC eluent was subject to positive ion nano‐flow electrospray analysis in an information‐dependent acquisition (IDA) mode. First, a TOF‐MS survey scans were acquired (*m*/*z* 350–1,500, 0.25 s) with the 20 most intense multiple charged ions (2+ to 5+; exceeding 200 counts per second) in the survey scan being sequentially subjected to MS/MS analysis. MS/MS spectra were accumulated for 100 ms in the mass range *m*/*z* 100–1,800 using rolling collision energy.

### 4.12 Data independent acquisition using SWATH mass spectrometry (SWATH‐MS)

Protein quantification using SWATH‐MS was performed as described by Kamath et al (https://pubs.acs.org/doi/10.1021/acs.jprot​eome.7b00561) with modification. Briefly, equal amounts of individual samples (approximately 2 μg) were separated over reverse‐phase linear gradient of 2 and 35% of mobile phase B over 88 min at a flow rate of 600 nl/min using the same LC and MS instruments as specified above with positive nano‐flow electrospray mode. In SWATH mode, first a TOF‐MS survey scan was acquired (*m*/*z* 350–1,500, 0.05 s), and then, the 100 predefined *m*/*z* ranges were sequentially subjected to MS/MS analysis. MS/MS spectra were accumulated for 30 ms in the mass range *m*/*z* 350–1,500 with rolling collision energy optimized for lowed *m*/*z* in *m*/*z* window +10%. To minimize instrument condition caused bias, SWATH data for each sample were acquired in a random order with one blank injection acquired between every sample injection.

### 4.13 Mass spectrometry data analysis

IDA‐MS data analysis and ion library generation are as follows. Respective IDA‐MS data files for brain and retina samples were consolidated and searched with ProteinPilot (v5.0, Sciex) using the ParagonTM algorithm in thorough mode. UniProt Mus musculus proteome database was used and searched using a tolerance of 2 missed tryptic cleavages. Fixed modifications were set for carbamidomethylation of cysteine. An Unused Score cutoff was set to 1.3 (95% confidence for identification). Resultant two data files were utilized as spectral/ion library for SWATH‐MS data analysis. Mass spectrometry data for both brain and retina samples were analyzed using a threshold of 1.2 fold change. Among the remaining list of proteins present in both retina and brain, specific AD proteins of interest were further highlighted (Table 1), and only if they were down‐regulated or up‐regulated significantly in either brain or retina of ADtg mice relative to WTs and restored either partially or fully to WT levels by GA treatment. A complete list of significantly identified proteins for brains (Table S1) and retina (Table S2) display fold change and comparisons between WT naïve and PBS control, as well as GA‐immunized and PBS control.

### 4.14 SWATH‐MS data analysis

Both Ion library and SWATH‐MS data files were imported into PeakView software 2.1 using the SWATH MicroApp 2.0 (SCIEX), and data were extracted using the following parameters: Top 6 most intense fragments of each peptide were extracted from the SWATH data sets (75 ppm mass tolerance, 5 min retention time window). Modified and shared peptides were excluded from quantification. After data processing, peptides (max 100 peptides per protein) with confidence >99% and FDR <1% (based on chromatographic feature after fragment extraction) were used for the quantitation. Cumulative protein areas from extracted ion chromatograms were exported to Excel for further analysis. The protein peaks areas were normalized to the total protein peak area of the respective sample and subjected to one‐sample *t* tests to compare relative protein peak areas between the respective sample groups. *t* test *p*‐value smaller than 0.05 and fold change ±1.2 was highlighted as differentially expressed proteins. Two approaches were considered for determining differential expression: ANOVA on the log‐transformed normalized protein peak areas of all samples and *t* test pairwise comparisons of pairs of specific samples. For the analysis of variance, proteins were deemed to be differentially expressed if the ANOVA p‐value was less than 0.05 and the maximum protein fold change exceeded 1.2.

### 4.15 Statistical analysis

Data were analyzed using GraphPad Prism 6.01 (GraphPad Software). A two‐tailed unpaired Student's *t* test was applied for analytic comparisons between two groups. Comparison of three or more groups was performed using one‐way ANOVA with Tukey's multiple comparison post‐test of paired groups. Analysis of two independent variables was performed using two‐way ANOVA with Sidak's post‐test. Correlation analysis was performed using Prism Pearson's tests. Results are expressed as means ± standard deviations (SDs) or means ± standard errors of the mean (SEMs) as indicated. A *p*‐value <0.05 was considered significant, and a *p*‐value <0.10 was regarded as a trend.

## CONFLICT OF INTEREST

Y.K., M.K.H., and K.L.B. are co‐founders and stockholders of NeuroVision Imaging, Inc., 1395 Garden Highway, Suite 250, Sacramento, CA 95833, USA.

## AUTHORS’ CONTRIBUTIONS

Study design: Maya Koronyo‐Hamaoui, Jonah Doustar, Yosef Koronyo; Acquisition of data: Jonah Doustar, Altan Rentsendorj, Tania Torbati, Giovanna C. Regis, Dieu‐Trang Fuchs, Julia Sheyn, Nazanin Mirzaei, Mitra Mastali, Yosef Koronyo, Maya Koronyo‐Hamaoui; MSD analysis: Mitra Mastali, Jonah Doustar, Yosef Koronyo, Julia Sheyn, Jennifer E. Van Eyk, Maya Koronyo‐Hamaoui; Mass spectrometry: Vivek K. Gupta, Stuart L. Graham, Nazanin Mirzaei; Analysis and interpretation of data: Jonah Doustar, Altan Rentsendorj, Giovanna C. Regis, Tania Torbati, Nazanin Mirzaei, Dieu‐Trang Fuchs, Julia Sheyn, Mitra Mastali, Yosef Koronyo, Maya Koronyo‐Hamaoui; Statistical analysis: Jonah Doustar, Altan Rentsendorj, Giovanna C. Regis, Tania Torbati, Dieu‐Trang Fuchs, Julia Sheyn, Nazanin Mirzaei, Mitra Mastali, Maya Koronyo‐Hamaoui; Drafting of manuscript: Maya Koronyo‐Hamaoui, Jonah Doustar, Tania Torbati, Giovanna C. Regis; Manuscript editing: Maya Koronyo‐Hamaoui, Jonah Doustar, Tania Torbati, Altan Rentsendorj, Giovanna C. Regis, Dieu‐Trang Fuchs, Mitra Mastali, Yosef Koronyo, Maya Koronyo‐Hamaoui; Discussion of results: Maya Koronyo‐Hamaoui, Jonah Doustar, Altan Rentsendorj, Tania Torbati, Giovanna C. Regis, Yosef Koronyo, Stuart L. Graham, Vivek K. Gupta, Keith L. Black, Prediman K. Shah, Nazanin Mirzaei; Study conception and supervision: Maya Koronyo‐Hamaoui. All authors read and approved the final manuscript.

## Data Availability

The datasets used and/or analyzed during the current study are available from the corresponding author on reasonable request. All experimental materials are commercially available and specified in the methods section.
