# Supplementary material for: Parallels between retinal and brain pathology and response to immunotherapy in old, late‐stage Alzheimer's disease mouse models
Source: Aging Cell. 2020 Oct 14;19(11):e13246. doi: 10.1111/acel.13246 (PMC7681044; doi:10.1111/acel.13246)
Supplement: Supplementary file 1 [file ACEL-19-e13246-s001.pdf]

SUPPLEMENTAL DATA

Parallels between retinal and brain pathology and response to immunotherapy in old, late-stage mouse AD models

Jonah Doustar[1], Altan Rentsendorj[1]*, Tania Torbati[1,2]*, Giovanna C. Regis[1]*, Dieu-Trang Fuchs[1], Julia Sheyn[1], Nazanin Mirzaei[1], Stuart L. Graham[3,4], Prediman K. Shah[5], Mitra Mastali[6,7], Jennifer E. Van Eyk[6,8,9], Keith L. Black[1], Vivek K. Gupta[10], Mehdi Mirzaei[3,10,11], Yosef Koronyo[1], Maya Koronyo-Hamaoui[1,6]♣

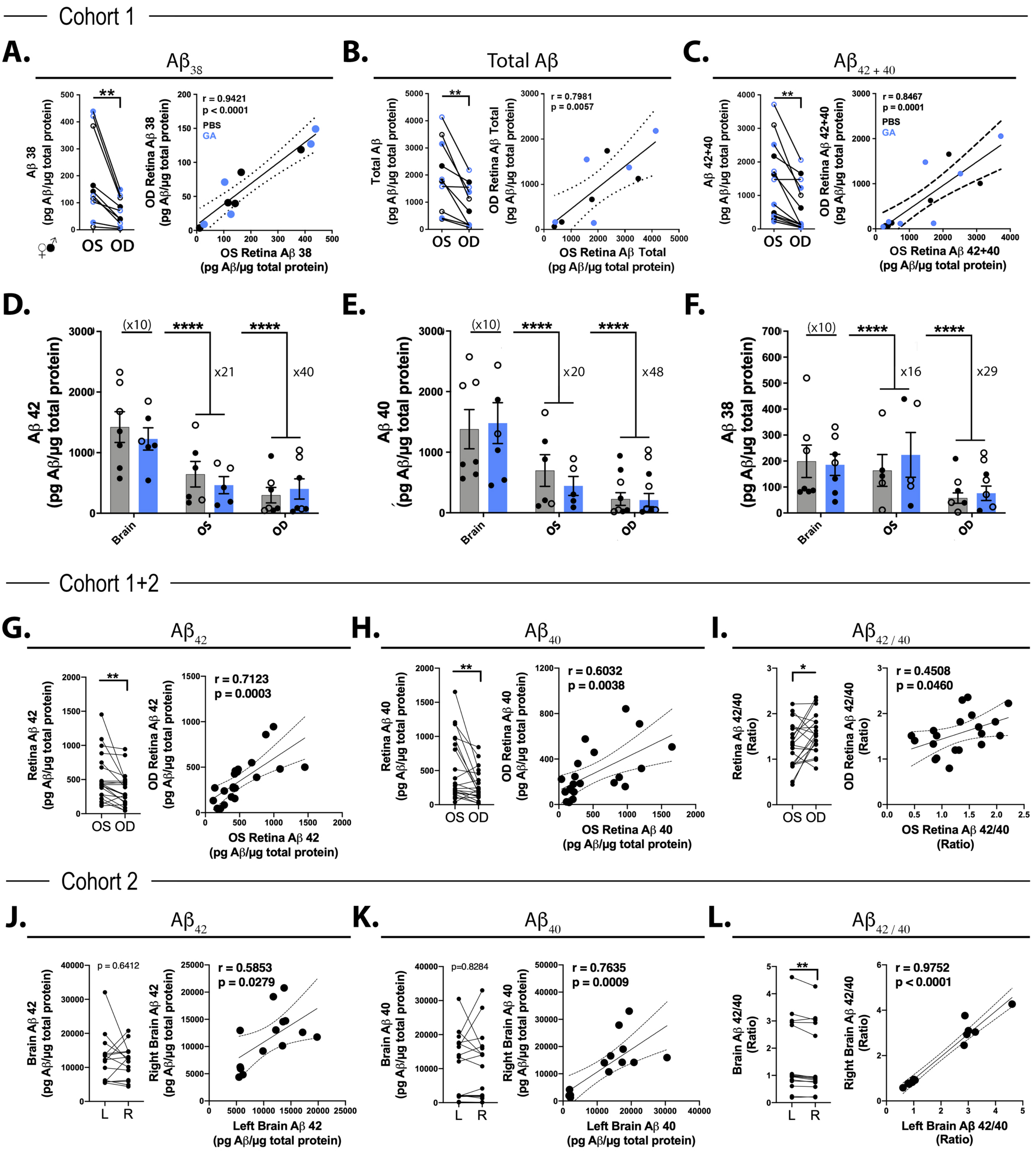

Suppl. Fig 1. Extended data on retinal and cerebral Aβ alloform levels, lateralization, and correlations in old ADtg mice, including after GA immunotherapy.

Supplemental Figure 1. Extended data on retinal and cerebral Aβ alloform levels, lateralization, and correlations in old ADtg mice, including after GA immunotherapy. (**A-C**) MSD analysis of (**A**) $Aβ_{1-38}$, (**B**) total $Aβ_{1-42}$, $Aβ_{1-40}$, and $Aβ_{1-38}$ peptides, and (**C**) sum of $Aβ_{42}$ and $Aβ_{40}$ in OS vs. OD retinae of PBS-control and GA-treated ADtg mice (Cohort 1; n = 14-10). Paired analysis (left graphs) and Pearson's *r* correlations (right graphs) for Aβ levels in the OS retina vs. the OD retina in each individual mouse (n = 5 – 7 mice per group; GA, blue dots, PBS, black dots). (**D-F**) Group analyses of PBS-control vs. GA-treated experimental groups for cerebral and retinal (**D**) $Aβ_{1-42}$ (**E**), $Aβ_{1-40}$, and (**F**) $Aβ_{1-38}$ levels (brain units are x10 pg per μg). (**G-I**) Extended MSD data for Retinal $Aβ_{1-42}$, $Aβ_{1-40}$ levels and ratios in old ADtg mice (Cohort 1 and 2; n = 21-20). Differential levels (left graphs) of (**G**) $Aβ_{1-42}$, (**H**) $Aβ_{1-40}$, and (**I**) $Aβ_{42/40}$ ratio, and correlations (right graphs), between OS and OD retinae, as determined by paired student *t-test* and Pearson's (*r)* coefficient analyses. (**J-L**) Extended MSD data for Brain Aβ levels in old ADtg mice (Cohort 2; n = 14-15). Differential levels (left graphs) of (**J**) $Aβ_{1-42}$, (**K**) $Aβ_{1-40}$, and (**L**) $Aβ_{42/40}$ ratio, and correlations (right graphs), between L and R posterior brains, as determined by paired student *t-test* and Pearson's (*r)* coefficient analyses. Graphs display individual data point for each mouse, with bar graphs also indicating group mean and standard error of mean (SEM) values. Mouse sex is designated as filled circles for males and open circles for females (gender not shown in correlation graphs). * $P < 0.05$, ** $P < 0.01$, assessed by paired student t-test for two-group comparisons, and a two-way ANOVA with Sidak's post-test for group analysis for brain and retinal tissues.

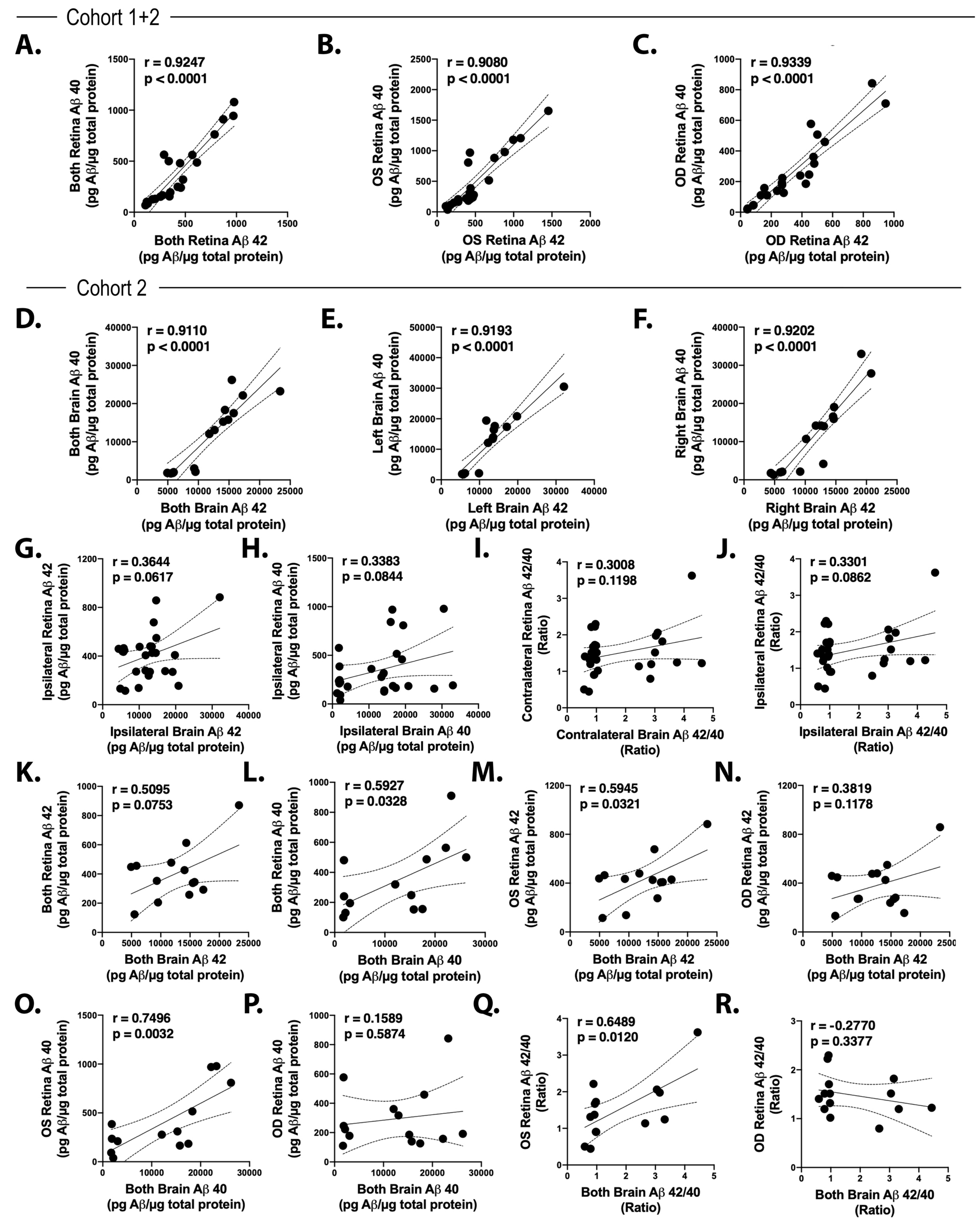

Suppl. Fig 2. Relationship between amyloidogenic alloforms in the same retinal and brain location and retino-cerebral associations of Aβ burden in old ADtg mice.

Supplemental Figure 2. Relationship between amyloidogenic alloforms in the same retinal and brain location and retino-cerebral associations of Aβ burden in old ADtg mice. (**A-C**) Pearson's *r* correlation analyses of intra retinal-tissue $Aβ_{1-42}$ and $Aβ_{1-40}$ alloforms and their ratio within (**A**) both, (**B**) OS, and (**C**) OD retinae of old ADtg mice (Cohorts 1 and 2; n = 22-21). (**D-F**) Pearson's *r* correlation analyses of intra-posterior brain tissue $Aβ_{1-42}$ and $Aβ_{1-40}$ alloforms and their ratio within (**D**) both, (**E**) left brain, and (**F**) right brain of old ADtg mice (Cohort 2). (**G-R**) Extended data of Pearson's *r* correlations between retinal and cerebral $Aβ_{1-42}$ and $Aβ_{1-40}$ alloform levels and their ratio in old ADtg mice (Cohort 2; n = 14-13). (**G-H**) Association between ipsilateral retinal and brain tissues (OD retina to right brain or OS retina to left brain) for levels of (**G**) $Aβ_{1-42}$ and (**H**) $Aβ_{1-40}$. (**I-J**) Associations between contralateral (OD retina to left brain or OS retina to right brain) and ipsilateral tissues for $Aβ_{42/40}$ ratios. (**K-L**) Associations between mean $Aβ_{42}$ and $Aβ_{40}$ levels in both posterior brain hemispheres and retinae. (**M-N**) Associations between both posterior brains against (**M**) OS and (**N**) OD retinae for $Aβ_{42}$ levels. (**O-P**) Pearson's *r* correlation analyses of $Aβ_{40}$ levels from both posterior brains against (**O**) OS and (**P**) OD retinae. (**Q-R**) Pearson's *r* correlations of $Aβ_{42/40}$ ratios from both posterior brains against (**Q**) OS and (**R**) OD retinae. Graphs display individual mouse data points.

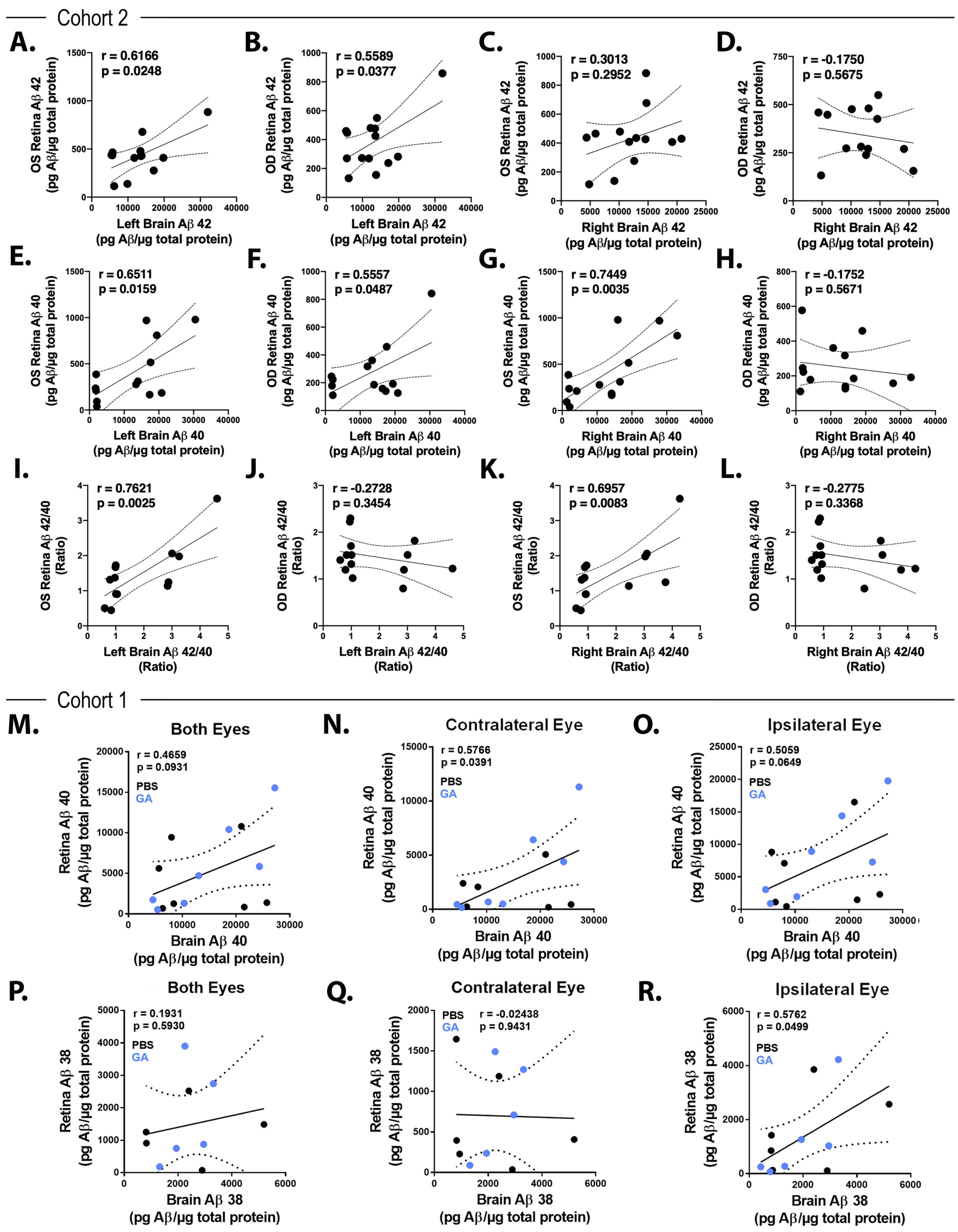

Suppl. Fig 3. Expanded data on retino-cerebral associations of Aβ burden in old ADtg mice and following GA treatment.

Supplemental Figure 3. Expanded data on retino-cerebral associations of Aβ burden in old ADtg mice and following GA treatment. (**A-L**) Pearson's *r* correlation analyses of Aβ alloforms in specific retinal vs. cerebral locations in old ADtg mice (Cohort 2; n = 14-15). (**A-D**) Associations between retinal and brain $Aβ_{42}$ levels: (**A**) OS retina vs left brain, (**B**) OD retina vs left brain, (**C**) OS retina vs right brain, and (**D**) OD retina vs right brain. (**E-H**) Associations between retinal and brain $Aβ_{40}$ levels: (**E**) OS retina vs left brain, (**F**) OD retina vs left brain, (**G**) OS retina vs right brain, and (**H**) OD retina vs right brain. (**I-L**) Associations between retinal and brain $Aβ_{42/40}$ ratios; (**I**) OS retina vs left brain, (**J**) OD retina vs left brain, (**K**) OS retina vs right brain, and (**L**) OD retina vs right brain. (**M-R**) Extended data for Figure 2N-Q. Pearson's *r* correlations of retinal vs. cerebral Aβ levels in Cohort 1 old ADtg mice following GA immunization (n = 14 mice). Associations in levels of retinal and brain $Aβ_{40}$ (**M-O**) and $Aβ_{38}$ (**P-R**) (n = 10 mice). Graphs display individual mouse data points.

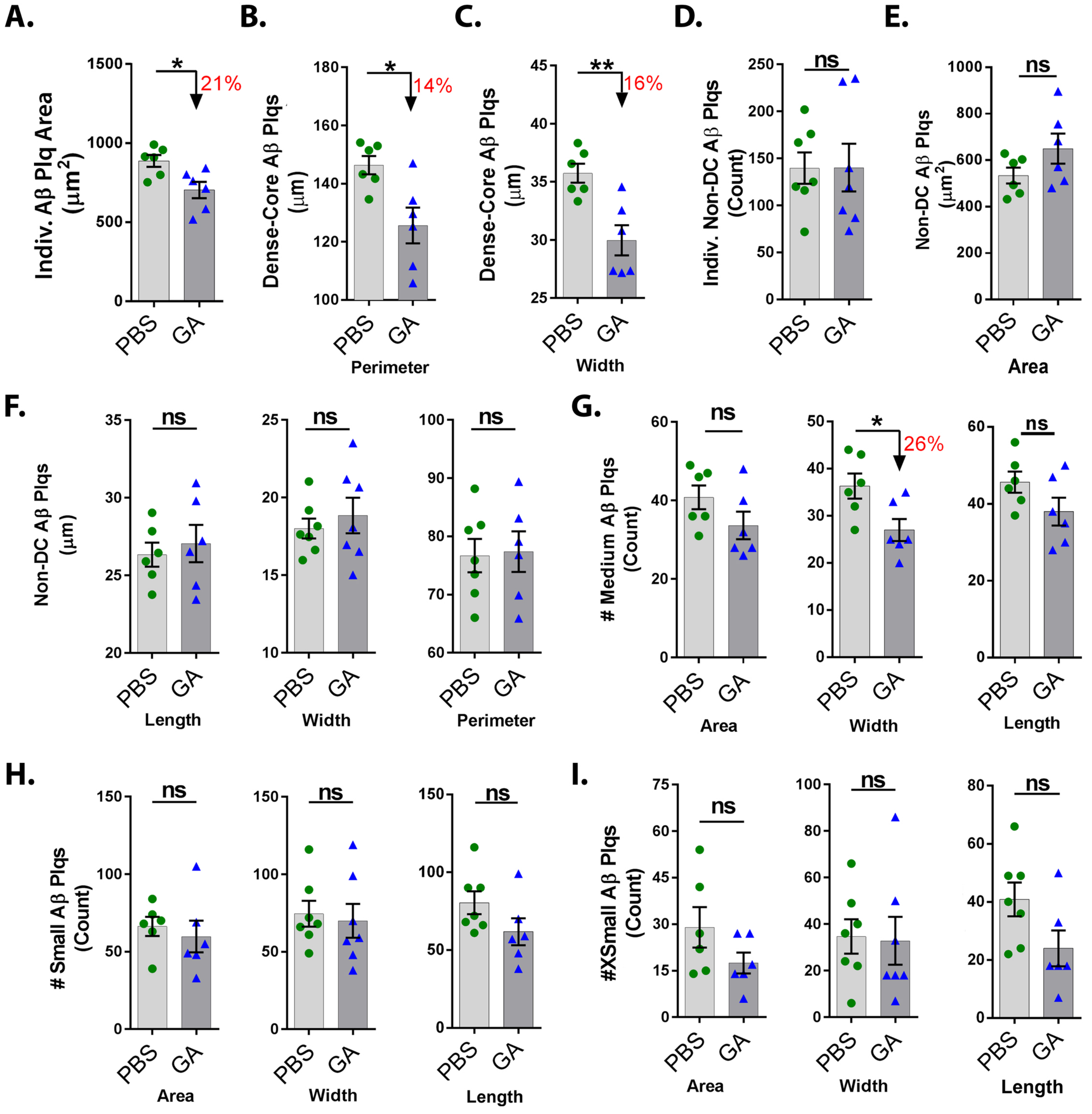

Suppl. Fig 4. Expanded data on effects of Immunomodulation on certain plaque size, morphology and subtype in old ADtg mice.

Supplemental Figure 4. Expanded data on effects of immunomodulation on certain plaque size, morphology and subtype in old ADtg mice. (**A-I**) Quantitative IHC analyses of Aβ plaque count and area by category and subtype in GA-immunized vs. PBS-control mice from Cohort 1 (n = 6-7 mice per group). Bar graphs between PBS-control and GA-immunized groups indicate (**A**) individual Aβ plaque area, (**B**) measurement of perimeter length of dense-core Aβ plaques, (**C**) measurement of dense-core Aβ plaque width, as well as (**D**) Non-dense core (Non-DC; diffuse) Aβ plaque total count, and (**E**) Non-dense core Aβ plaque area. (**F**) Measurement of non-DC Aβ plaques between treatment groups including length (left), width (center) and perimeter (right). (**G**) Medium Aβ plaque average count for all three measurements: area (left), width (center), length (right), (**H**) Small Aβ plaque average count for all three measurements: area (left), width (center), length (right), (**I**) Extra-small (X-small) Aβ plaque average count for all three measurements: area (left), width (center), length (right), Bar graphs indicate mean, standard error of mean (SEM), and individual data points. * $P < 0.05$, ** $P < 0.01$, by unpaired student t-test.

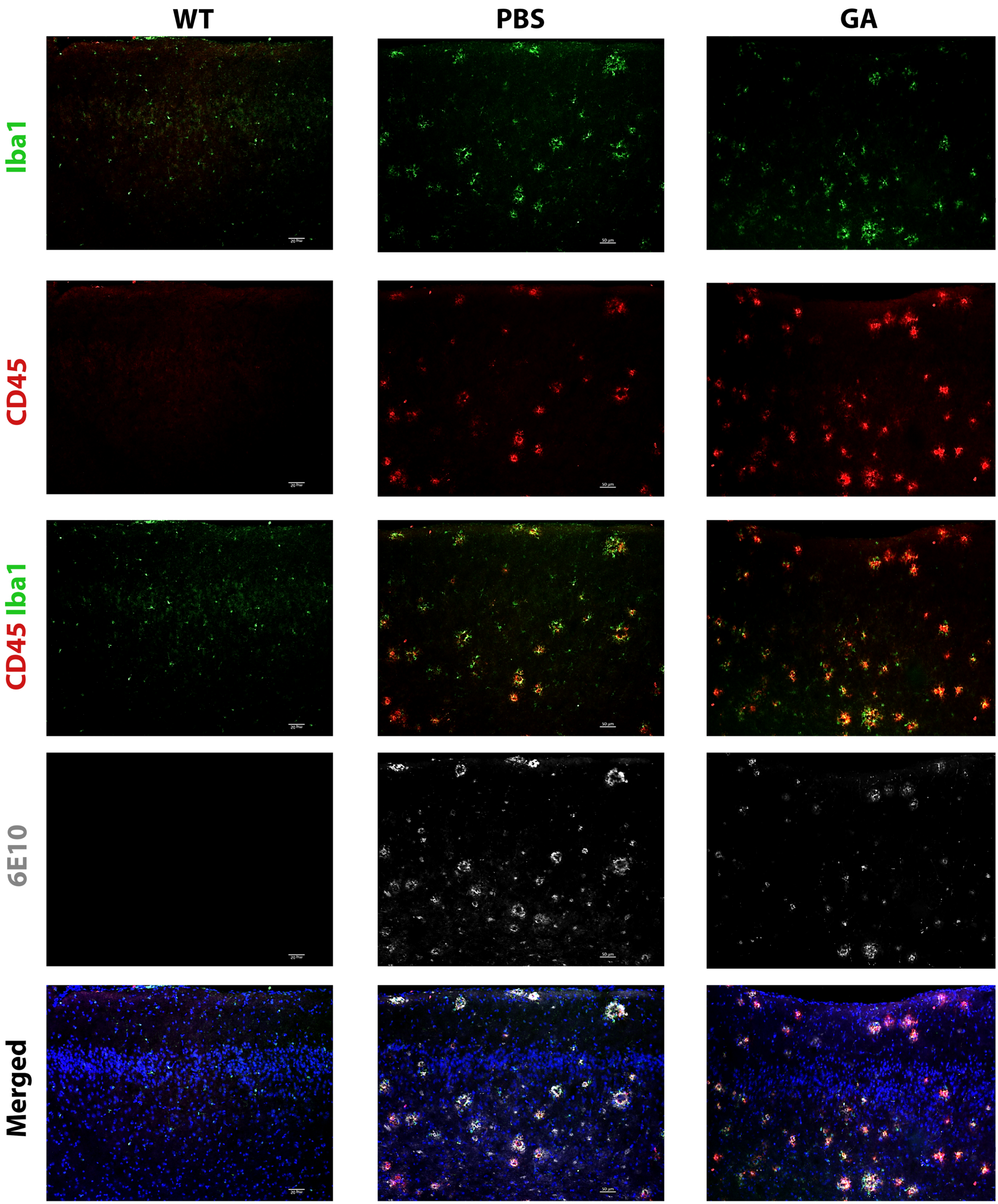

Suppl. Fig 5. Effects of GA-immunization on infiltrating immune cell populations in the Entorhinal Cortex.

Supplemental Figure 5. Effects of GA-immunization on infiltrating immune cell populations in the Entorhinal Cortex. Representative micrographs of Iba1+ (green), CD45 (red), CD45/Iba1+ co-labelling, 6E10 (white; Aβ plaque), and merged composite images including DAPI stained nuclei (blue). Experimental groups include WT-naïve littermates (left), PBS-control (middle), and GA-immunized mice (right). Scale bars = 50 μm.

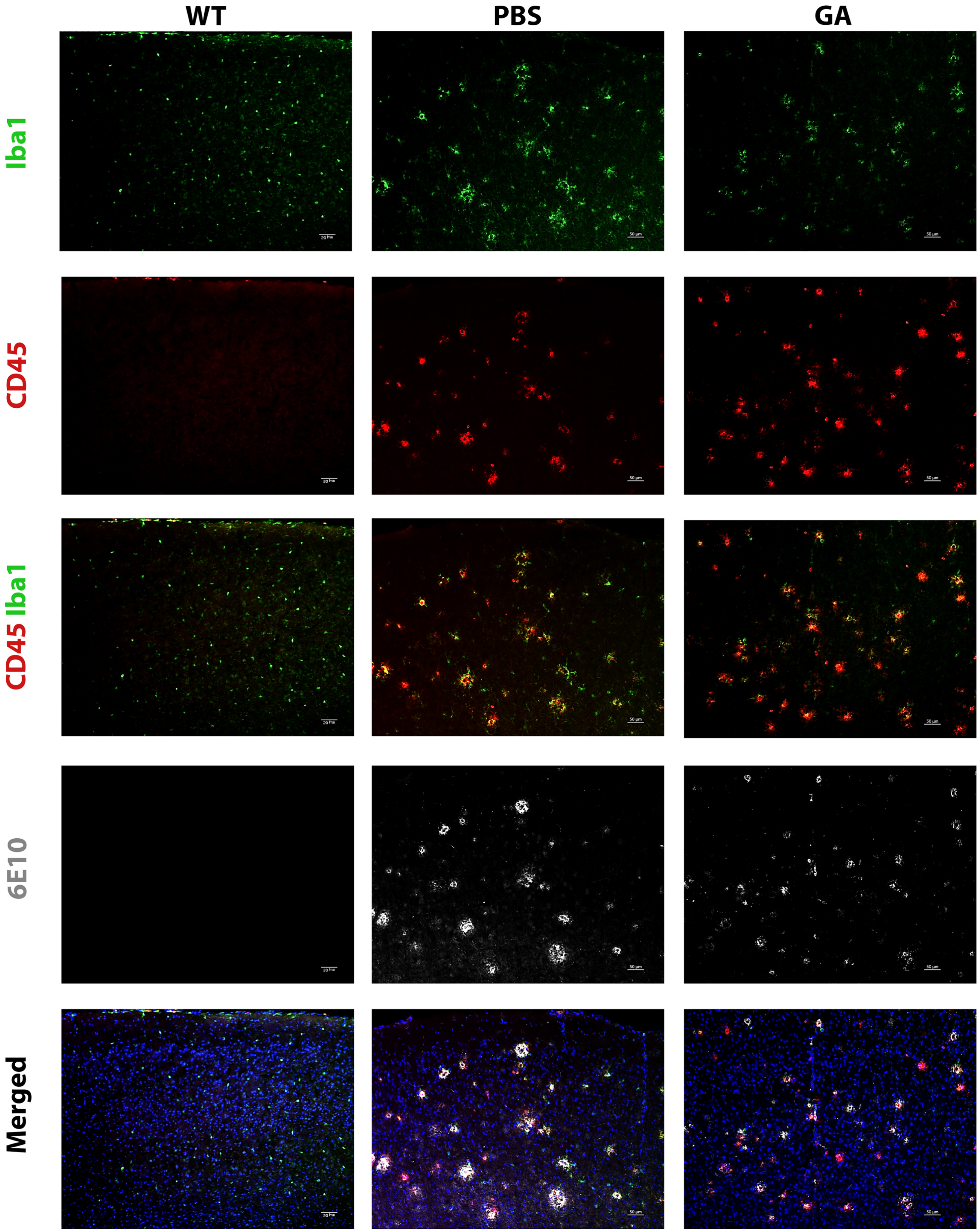

Suppl. Fig 6. Effects of GA-immunization on infiltrating immune cell populations in the Cingulate Cortex.

Supplemental Figure 6. Effects of GA-immunization on infiltrating immune cell populations in the Cingulate Cortex. Representative micrographs of Iba1+ (green), CD45 (red), CD45/Iba1+ co-labelling, 6E10 (white; Aβ plaque), and merged composite images including DAPI stained nuclei (blue). Experimental groups include WT-naïve littermates (left), PBS-control (middle), and GA-immunized mice (right). Scale bars = 50 μm.

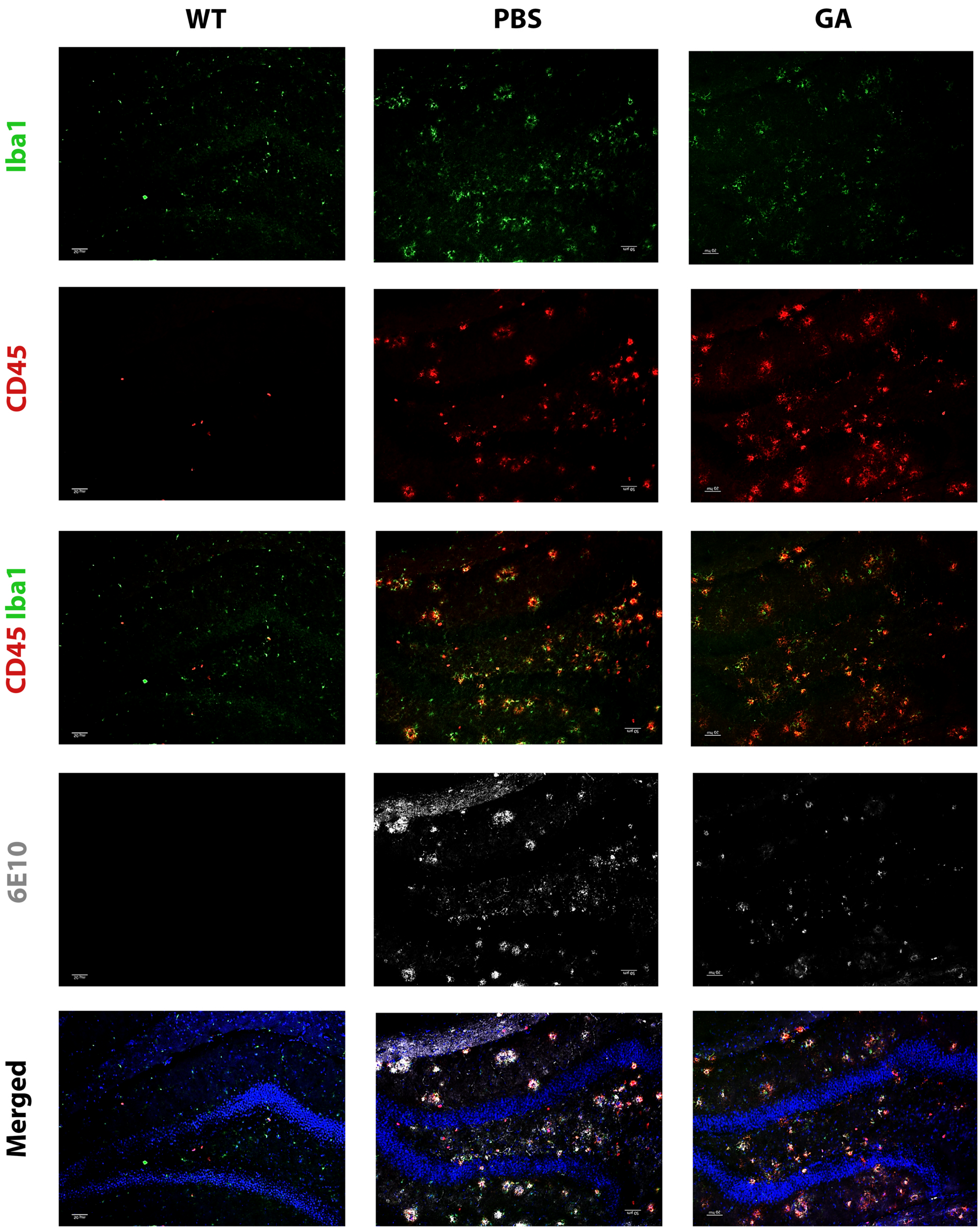

Suppl. Fig 7. Effects of GA-immunization on infiltrating immune cell populations in the Hippocampus.

Supplemental Figure 7. Effects of GA-immunization on infiltrating immune cell populations in the Hippocampus. Representative micrographs of Iba1+ (green), CD45 (red), CD45/Iba1+ co-labelling, 6E10 (white; Aβ plaque), and merged composite images including DAPI stained nuclei (blue). Experimental groups include WT-naïve littermates (left), PBS-control (middle), and GA-immunized mice (right). Scale bars = 50 μm.

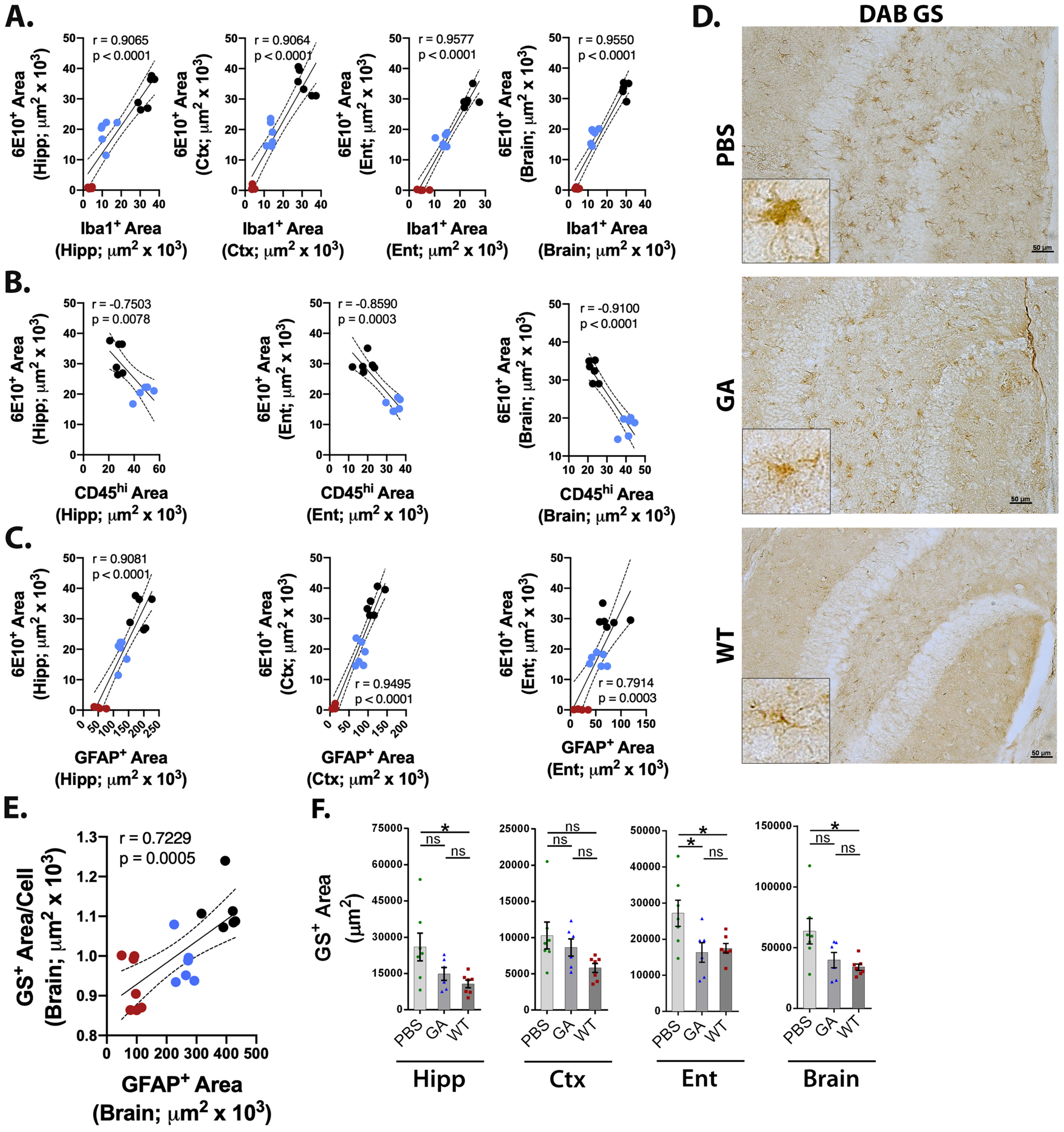

Suppl. Fig 8. Extended data on associations between inflammatory cells, plaque deposition and astrocytic GS expression.

Supplemental Figure 8. Extended data on associations between inflammatory cells, plaque deposition and astrocytic GS expression. (**A**) Pearson's *r* correlation analyses between $Iba1^+$ cell area and $6E10^+$ Aβ plaque area within Hipp, Ctx, Ent, and all three brain regions. (**B**) Pearson's *r* correlation analyses between $CD45_{hi}$ hematopoietic immune cells area and $6E10^+$ Aβ plaque area within Hipp, Ent, and all three brain regions. (**C**) Pearson's *r* correlation analyses between $GFAP^+$ astrogliosis area and $6E10^+$ Aβ plaque area within Hipp, Ctx, and Ent. (**D**) Hippocampal sections from all experimental groups stained for glutamine synthetase (GS), with peroxidase-DAB immunostaining. (**E**) Pearson's *r* correlation analysis between $GFAP^+$ astrocytic area and GS^+ immunoreactive area per GS^+ cell including all experimental groups (n = 19 mice). Black data points indicate PBS-control ADtg mice, blue data points indicate GA-immunized ADtg mice and red data points indicate WT-naïve littermates. (**F**) GS^+ area analysis between all experimental groups, within the Hipp, Ctx, Ent and all three brain regions (n = 6 – 7 mice per group). Bar graphs indicate the mean and the standard error of mean (SEM). * $P < 0.05$, ** $P < 0.01$, *** $P < 0.001$, **** $P < 0.0001$ assessed by one-way ANOVA with Tukey's post-test evaluation for three or more groups. Pearson's *r* correlation analysis was used to determine relations between two parameters.

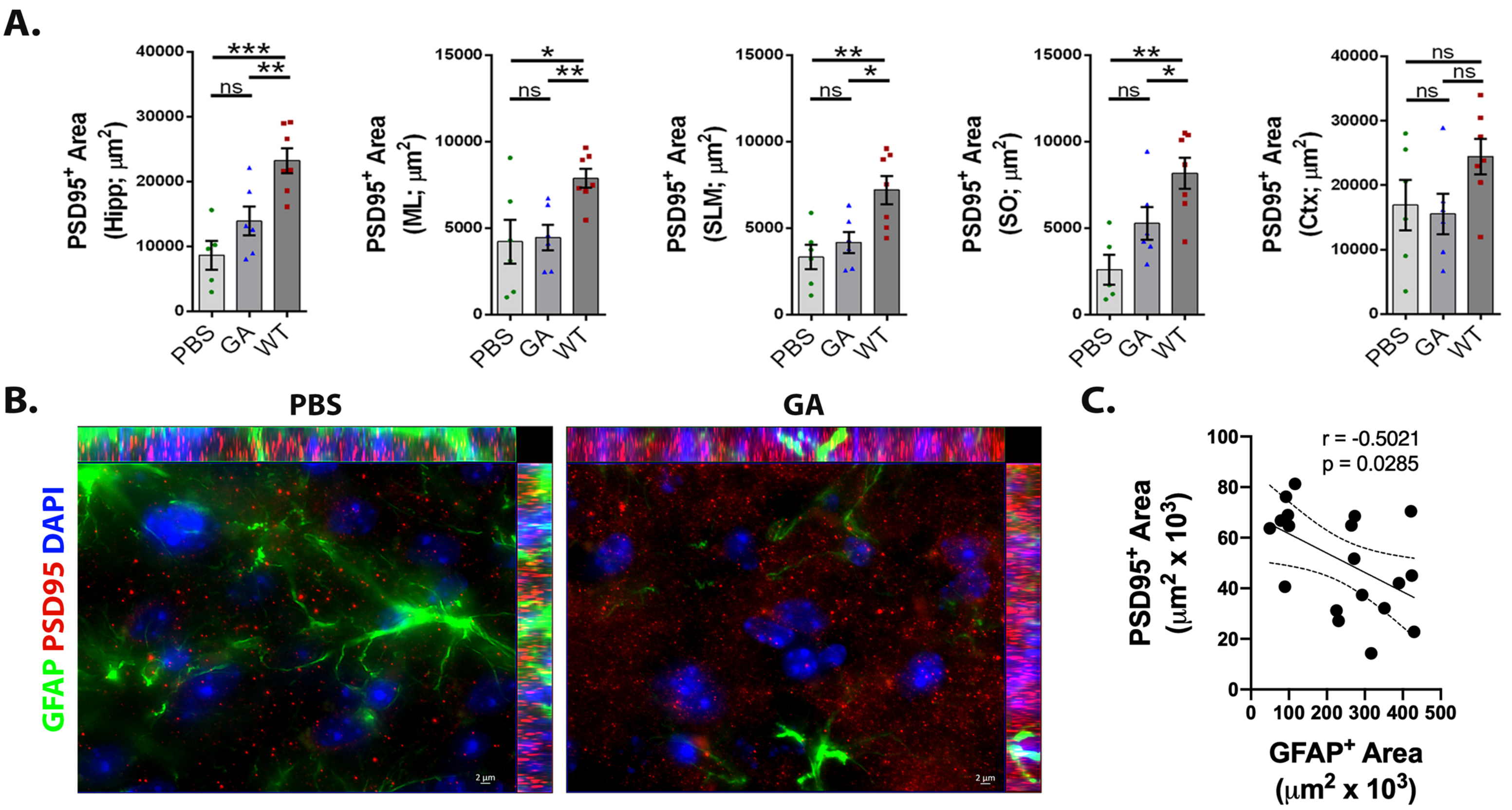

Suppl. Fig 9. Effects of GA immunization on post-synaptic density and its association with astrogliosis.

Supplemental Figure 9. Effects of GA immunization on post-synaptic density and its association with astrogliosis. (**A**) Immunohistochemical analysis of post-synaptic PSD95+ area within the Hipp, including the molecular layer (ML) of the dentate gyrus, the stratum lacunosum-moleculare (SLM), and the stratum oriens (SO) of cornu ammoni, along with the Ctx. Analysis between all experimental groups (n = 5 – 7 mice per group). (**B**) Representative high-magnification micrographs (x100) of GFAP+ astrocytes (green) and PSD95+ post-synaptic terminals (red) in GA-immunized vs. PBS-control ADtg mouse brains. Scale bar = 2 μm. (**C**) Pearson's *r* correlational analysis between the area of astrocytic reactivity (GFAP+) and post-synaptic (PSD95+) area, between all experimental groups (n = 19 mice). Bar graphs indicate the mean and the standard error of mean (SEM). * $P < 0.05$, ** $P < 0.01$, *** $P < 0.001$ assessed by one-way ANOVA with Tukey's post-test evaluation for three or more groups. Pearson's *r* correlation analysis was used to determine relations between two parameters.
